# Effects of Investigational Moisturizers on the Skin Barrier and Microbiome following Exposure to Environmental Aggressors: A Randomized Clinical Trial and Ex Vivo Analysis

**DOI:** 10.3390/jcm12186078

**Published:** 2023-09-20

**Authors:** Dan-Qi Wang, Xi Li, Ru-Yi Zhang, Chao Yuan, Bo Yan, Philippe Humbert, Zhe-Xue Quan

**Affiliations:** 1 Ministry of Education Key Laboratory for Biodiversity Science and Ecological Engineering, Fudan Microbiome Center, Institute of Biodiversity Science, School of Life Sciences, Fudan University, Shanghai 200437, China; 18110700020@fudan.edu.cn (D.-Q.W.); 16210700125@fudan.edu.cn (R.-Y.Z.); 2 Translational Science Asia Pacific, Shanghai Technology and Research Center, Johnson & Johnson (China) Ltd., Shanghai 200245, China; byan7@its.jnj.com; 3 Skin and Cosmetic Research Department, Shanghai Skin Disease Hospital, Shanghai 200443, China; dermayuan@163.com; 4 Department of Dermatology, Clinical Investigation Center, Besancon University Hospital, 25030 Besancon, France; philippehumbert25@gmail.com; 5 IRDR ICoE on Risk Interconnectivity and Governance on Weather/Climate Extremes Impact and Public Health, Fudan University, Shanghai 200437, China

**Keywords:** skin microbiome, moisturizer, environmental aggressors, postbiotics

## Abstract

The skin microbiota barrier participates in skin barrier function in addition to the physical, chemical, and immunological protective barriers, and is affected by environmental aggressors and skincare regimens. To better understand the exact effects of real-life environmental conditions on the skin and determine the protective methods, this study investigates the effects of three topical cosmetic moisturizers (water gel moisturizers with/without yeast extract (Moisturizers K and C) and a thick-emulsion cream moisturizer (Moisturizer L)) on clinical and skin microbiome endpoints in the presence of environmental aggressors during an 8-week, randomized controlled, triple-blind clinical trial with 110 participants, and molecular- as well as biomarker-level endpoints on ex vivo skin explants after exposure to simulate urban environmental conditions. The results show that all moisturizers are well-tolerated and improve skin barrier function and surface moisture content from the baseline, and the improvement is maintained at the last analysis point (3 days after trial completion). Compared with the untreated control areas (samples taken from the upper chest), treatment with Moisturizer K prevented a reduction in bacterial and fungal richness, and increased the change ratio of the relative abundance of commensal bacteria, such as *Staphylococcus epidermidis* and *Ralstonia*, at the treated sites (samples taken from the forehead). Moreover, Moisturizer K-treated ex vivo skin explants had higher levels of caspase 14 (a marker of skin barrier function), collagen I, and elastin (structure components), and lower levels of aryl hydrocarbon receptor (AHR; activated by air pollutants) and interleukin-6 (IL-6) than those in explants treated with other moisturizers and in the untreated areas of the skin. These results suggest that a skin postbiotic moisturizer with yeast extract supports the regulation of the skin’s microbiome balance and may provide a holistic barrier (involving skin microbiome, physical, chemical, and immune barriers) to protect the skin against environmental aggressors.

## 1. Introduction

The skin is the largest organ of the human body; it interfaces with the external environment and adapts to it, while protecting it against environmental aggressors. In addition to the skin’s outermost physical barrier (the stratum corneum), a microbial barrier of commensal and transiting microflora protect the skin from mechanical and chemical intrusions [1]. The skin microbiome plays a critical role in maintaining human health and the disruption of its balance can lead to skin diseases [2,3]. The skin barrier also includes chemical and immunological protective actions, such as antimicrobial peptides released by keratinocytes and the presence of hematopoietic cells, including T, natural killer, and mast cells [1,4]. Collectively, these cells and microbiota create a dynamic skin barrier.

Exposure to environmental aggressors, such as particulate matter (pm), UV, temperature and humidity, and wind, or improper skincare habits, such as over-cleansing, may damage the protective envelope of the stratum corneum and impair its physiological balance, causing water loss and consequent skin dryness, flaking, or cracking [5,6,7]. Environmental stress can also significantly reduce the synthesis of collagen I (associated with skin strength and firmness) and elastin (associated with skin softness and elasticity) [7]. Adequate hydration and a healthy trans-epidermal water loss (TEWL) are also fundamental to maintaining intact skin protection, and the routine use of topical skin emollients aims to hydrate the skin and promote skin repair to protect it against negative physical stimuli [8,9]. Skin emollients work primarily in three manners: supplementing the water content by applying hygroscopic ingredients, such as urea, hyaluronic acid, and sorbitol; reducing TEWL by applying occlusive substances, such as petroleum, mineral oil, or beeswax; and forming a shield to smoothen the irregular surface caused by the shedding of corneocytes (i.e., glycerin) [9,10]. Skin natural moisturizing factors (NMFs) located in the stratum corneum are responsible for the absorption and retention of water. The emollient formulation along with the functional technology, such as hydration, postbiotic, deliver system, etc., may influence the penetration and/or efficacy of a particular product by impacting NMFs [11,12]. To achieve sensory and efficacy benefits, a cosmetic moisturizer was formulated with a delicate balance of water and oil to prevent water loss and with additional functional technologies to boost skin hydration to fulfill the sensory preference based on skin types.

Components of personal skincare products may remain on the skin for weeks and may impact the skin microbiota [13]. Moreover, new protocols for cosmetic antipollution efficacy evaluation have been reported [14,15]. However, the impact of external agents, as well as environmental aggressors, on the barrier function of the skin and its microbiome (including *Staphylococcus* and *Streptococcus*) is not understood well. This study clinically evaluates the impact of real-life environmental aggressors on the skin microbiome and the protection conferred by the consistent application of three types of non-prescription moisturizing formulations. The effect of the investigational moisturizers is also assessed at molecular and biomarker levels on skin explants after exposure to simulated city environmental conditions in summer. Some results from this study were presented at the American Academy of Dermatology meeting as a poster in 2021 [16]; in this manuscript, we present the entirety of the analysis results.

## 2. Materials and Methods

### 2.1. Participants

This study investigated several hydration-related clinical endpoints (skin moisturization, barrier function, and translucency) and the shift in skin microbiome profiles of skin areas treated with three experimental moisturizers compared with those in untreated control sites. Participants in the trial were exposed to real-life environmental conditions in a major city (Shanghai, China).

The study was conducted from May (late spring) to July (summer) during the humid subtropical season. Environmental conditions at that time were warm/windy with light pollution in late spring, transitioning to ‘plum rain’ (rainy/cloudy, hot, and humid), and finally shifted to hot and sunny in the summer, when the participants were exposed to indoor air conditioning (Figure S1).

Eligible participants were Shanghai-based Chinese females between 18 and 40 years of age with self-assessed dry, but healthy, facial skin based on the skin-type self-perceived questionnaire. Participants showed premature aging signs (i.e., fine lines, dullness) and a Corneometer^®^ CM 825 (Courage + Khazaka electronic GmBH, Köln, Germany) score of ≤35 arbitrary units (a.u.) for both cheeks. Participants were required to stay in Shanghai during the study and continue with their daily city lives, experiencing usual levels of exposure to the environment (e.g., outdoor activities). Participants were sorted into four lifestyle categories: office worker, housewife, student, and outdoor worker. Any participant that did not fall into these categories was classified as ‘other’.

### 2.2. Study Design and Protocol

The study was designed as a single-center; participant, investigator, and assessor triple-blind; and 1:1:1 randomized controlled study. Randomization was stratified to ensure an even distribution between groups among occupational lifestyles and Corneometer^®^ scores.

After enrollment, the participants underwent a 3-day wash-out period, during which each participant cleaned their face and upper chest area twice daily with water only. During the 8-week study period, the participants washed their face twice a day (morning and evening) with 1 mL of a standard milk cleanser and warm water, before applying 1 mL of their assigned moisturizer to their whole face. For each participant, the left or right cheek was randomly chosen as the evaluation area, and the middle upper chest area, which had the same environmental exposure, was designated as the untreated control.

Participants were assessed at the baseline (i.e., on completion of the 3-day wash-out period, prior to moisturizer application), 2–4 h (3 H), and 8 h (8 H) after moisturizer applications. Participants were evaluated by a certified, well-trained, and experienced dermatologist with clinical grading capability at the Shanghai Skin Disease Hospital (the study site) at screening, baseline, 1 week (1 W), 4 weeks (4 W), 8 weeks (8 W), and 8 weeks + 3 days (8 W + 3 D, i.e., a regression period visit 3 days after the last moisturizer application).

### 2.3. Investigational Products

Participants were randomized to apply three products, a thin water gel-like format with kiwi-derived yeast extract, which was patented postbiotic technology (water gel with yeast extract) [17]; a thin gel-like format (water gel); and a thicker-emulsion cream format (extra-dry emulsion), and these three products were named as Moisturizers K, C, and L, respectively (as the increase in skin water content by these products was confirmed in this clinical study). All products were from the Neutrogena Hydro Boost line. Details about the composition of each moisturizer are presented in Table S1.

To preserve study blinding, all products were supplied in artwork-free packaging (see Supplementary Materials for detailed information). The use of any other cosmetic products, sunscreen, make-up, or topical treatment on the face and upper chest area was not permitted during the study period.

### 2.4. Endpoints and Assessments

The primary endpoint was the change in skin surface moisture content for each moisturizer group, which was evaluated by measuring the skin surface capacitance using a Corneometer^®^ and reported as the average of five measurements. Skin barrier function was assessed by TEWL (trans-epidermal water loss) using Tewameter^®^ TM300 (Courage + Khazaka electronic GmBH, Köln, Germany), and the ratio of TEWL and the Corneometer^®^ value (T/C ratio) were calculated to estimate the relative trans-epidermal water loss per skin water content. Skin translucency was assessed by a Translucency Meter (TLS850, Dia-Stron Limited, Hampshire, UK), and an average of two readings was taken to report the K value. These endpoints were defined as per formula design benefits and related traditional skin physiology measures, which were also used in a previous publication by the team [18,19].

Clinical grading and adverse events were recorded as reported by an experienced dermatologist. Clinical study details, including inclusion and exclusion criteria, triple-blind design, randomization details, and the qualification and training of dermatologists can be found at: https://clinicaltrials.gov/ct2/show/study/NCT03264677?term=NCT03264677&draw=2&rank=1 (accessed on 20 July 2023).

The data on environmental aggressors (temperature, humidity, wind, ultraviolet (UV), and air quality (defined as particulate matter (PM) 2.5; mass concentration (μg/m^3^) of particles with aerodynamic diameter < 2.5 μm)) were collected daily during the study period from an official website for local weather in Shanghai (Figure S1) [20].

### 2.5. Statistical Analysis

A minimum sample size of 35 participants per group was considered sufficient to detect significant within-group differences in the clinical assessments during the study period compared with the baseline based on historical experience with a similar type of study, cosmetic industry guidance, and group standards of cosmetic efficacies. Additional testing was performed to the detect significant differences in parameters between groups (baseline and no treatment control-adjusted), within the group multi-comparison at the baseline, and during each study visit. A clinical statistical analysis was conducted using SPSS^®^ Statistics version 19.0 (IBM, New York, NY, USA). Standard normality and equality of variance tests were performed for each parameter–clinical grade and for instrumental readings to determine whether the data were parametric and/or non-parametric. Paired/independent *t*-tests (two-sided test with alpha set to 5%) and ANOVA (LSD/Dunnett’s comparison) and Wilcoxon test (for non-parametric data) were performed for each parameter to determine the significant differences in subsequent readings between different locations and groups.

### 2.6. Exploratory Endpoints of Skin Microbiome

To assess the change in the skin microbiome after moisturizer application and/or environmental aggressors, skin microbiome samples were collected from 97 participants in the study and analyzed according to the lifestyle categories mentioned above.

The skin microbiome was assessed using samples obtained from the skin. Swabs were rubbed firmly on 2 × 4 cm^2^ areas of skin on the forehead above the eyebrow (treated area) and 2 × 4 cm^2^ areas of skin on the upper chest/neckline above the inner end of the collarbone (non-treated area; Figure S2). Skin microbiome sampling was performed at the baseline, 4 W, and 8 W into the study, and DNA was extracted from 582 skin surface swab samples using the PowerSoil^®^ DNA Isolation Kit (Qiagen, Valencia, CA, USA) following the manufacturer’s instructions, with slight modifications. Polymerase chain reaction (PCR) amplification for the bacterial 16S rRNA hypervariable V1–V2 region and fungal internal transcribed spacer 1 (ITS1) region was performed. Post-sequencing data analysis and a taxonomic classification were performed following the Quantitative Insights Into Microbial Ecology (QIIME) version 1.8 pipeline [21] (see Supplementary Materials for detailed methodology).

### 2.7. Microbiome Diversity Analysis

Microbial alpha diversities were determined using Chao 1, Shannon, and phylogenetic diversity indices, as well as the observed OTUs for bacteria. Chao 1, Shannon, and OTU numbers were also calculated for fungi to evaluate the richness and diversity within the communities. Beta diversity was used to demonstrate the differences between communities. Weighted UniFrac and Bray–Curtis distances were used to determine the community composition, while unweighted UniFrac and Binary Jaccard distances were used to assess community membership for the bacterial and fungal communities, respectively. Differences in the alpha diversity values and relative abundances of genera, as well as *Staphylococcus* and *Streptococcus* species, between sampling sites were identified using the Wilcoxon test. The Kruskal–Wallis test was used to assess the alpha diversity indices and relative abundances of genera, as well as species, with respect to the different sampling times, and participant lifestyles, reflected by their occupation categories.

To assess the effects of the three study moisturizers, the change rate of the relative abundance of different microbial taxa was calculated at 4 W and 8 W towards the baseline for both forehead samples and the upper chest controls, and then the comparison was performed using the Wilcoxon test. PERMANOVA was applied to measured intergroup differences among various sample community attributions. *p*-values were corrected using the false discovery rate method for pairwise comparisons and the Bonferroni method for multiple comparisons. All statistical analyses were performed using R (version 3.6.1) and QIIME (see Supplementary Materials for additional methodology).

### 2.8. Skin Explant Chamber Stimuli Model Assay

An ex vivo study was performed to investigate the protective effect of study moisturizers on human skin biopsies exposed to environment aggressors, including urban air pollutants and seasonal conditions. Skin explants from three Caucasian women undergoing abdominoplasty (aged 35–41 years old) were cleaned with a phosphate-buffered solution and the adipose tissue was removed before the samples were loaded into a hermetic cell (D-SKIN Cell^®^, developed by Professor Philippe Humbert’s research group; Figure S3) with two parts: a lower part (six wells with a water bath) and an upper part (hermetic cover). Donor skin samples were loaded to create two compartments, one on each side of the sample: a donor compartment (‘epidermal’ compartment) with an open surface of 3.14 cm^2^ applied to the upper side of the skin, and a receptor compartment (‘dermal’ compartment containing the receptor fluid) applied to the lower side of the tegument, comprising a 7 mL fixed-volume compartment.

The investigational moisturizers were applied directly to the skin explants from each donor with three repeats. Samples were exposed to simulated summer environmental conditions for an hour (temperature: 45 °C; humidity: 60%; UVA: 10 J/cm^2^; UVB: 0.4 J/cm^2^; PM2.5: 75 μg/m^3^; PM10: 150 μg/m^3^; and windspeed: 50 mL/m^3^/h). Following exposure, the skin explants were analyzed for several biomarkers within 2 h of moisturizer application. This involved immunostaining and microscopy for caspase 14 (PRS2509, Merck KGaA, Darmstadt, Germany), aryl hydrocarbon receptor (AHR; SAB1412326, Merck KGaA), collagen I (C2456, Merck KGaA), elastin (MOB230-100, Clinisciences, Koln, Germany), and IL-6 (CBL 2117, Clinisciences).

The statistical analysis of the ex vivo study was performed using Sigma^®^ statistical software (Systat Software Inc., San Jose, CA, USA). Analysis of variance (ANOVA) with one or two factors was performed and followed, if necessary, by a Fisher test. *p*-values < 0.05 were considered significant. Descriptive statistics were calculated for between- and within-group comparisons.

## 3. Results

### 3.1. Skin Clinical Parameters in Three Moisturizer Groups

One-hundred-and-twenty-two participants were recruited and randomized to the three intervention groups; of these, 110 participants completed the study. The number of participants for each lifestyle category was as follows: office worker (n = 65), housewife (n = 18), student (n = 11), outdoor worker (n = 11), and other (n = 5). Overall, the groups were well-balanced for age, lifestyle, and baseline skin type (Table 1). The data for the environmental conditions during the study period are shown in Figure S1.

**Figure 1:**
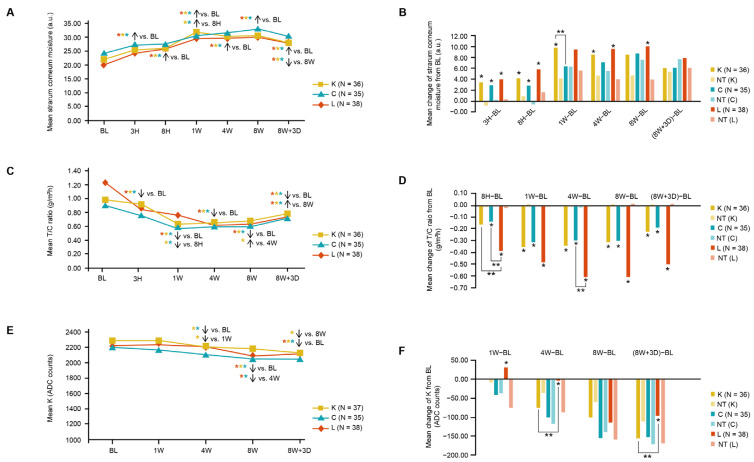
Clinical efficacy parameters for the three investigated moisturizer groups and untreated areas across the 8-week randomized, controlled, and triple-blind clinical trial. Comparison of skin stratum corneum moisture content (measured using a Corneometer^®^) between the (**A**) time points of moisturizer groups and (**B**) moisturizers/NT; comparison of skin barrier function (T/C) between the (**C**) time points of moisturizer groups and (**D**) moisturizers/NT; comparison of skin translucency (K value; a lower K value indicates increased skin translucency) between the (**E**) time points of moisturizer groups and (**F**) moisturizers/NT. Abbreviations: 8 H, 8 h; 1 W, 1 week; 4 W, 4 weeks; 8 W, 8 weeks; 8 W + 3 D, 8 weeks + 3 days (3 days after treatment is stopped); ADC, analog-to-digital converter (light intensity count); a.u., arbitrary unit; BL, baseline; K, water gel with yeast extract; C, water gel; L, extra-dry emulsion; T/C, ratio of trans-epidermal water loss and surface moisture content measured by Corneometer^®^ (which indicates the relative skin barrier function to skin water content). * *p* < 0.05. ** *p* < 0.01. *p*-values are derived using the *t*-test and ANOVA.

All moisturizers significantly increased skin moisture at 8 W versus the baseline (primary endpoint), as well as all other study time points versus the baseline and at 8 W versus 8 W + 3 D (*p* < 0.05; Figure 1A). Compared with the untreated control sites, all moisturizers showed long-lasting skin hydration. The mean change in skin surface moisture from the baseline was significantly improved with Moisturizers K (1 W, 4 W) and L (4 W, 8 W). However, no significant difference was observed between the three moisturizers at any time point other than at 1 W, where Moisturizer K improved skin surface moisture significantly more than Moisturizer C (Figure 1B). During the 8-week study period and environmental challenge, the untreated control areas of the skin showed a significantly worse skin barrier function (TEWL), which was consistent with the clinical results of decreased microbiome richness (see subsequent section).

All moisturizers yielded significant decreases in the T/C ratio (which indicated a skin barrier function relative to the skin water content [22]) at 8 H, 1 W, 4 W, 8 W, and 8 W + 3 D, compared with that at the baseline (Figure 1C), and the T/C ratio was significantly lower at 8 W versus 8 W + 3 D. Compared with the control skin areas, all moisturizers resulted in significantly greater decreases in T/C, compared to the baseline, at 1 W, 4 W, 8 W, and 8 W + 3 D (Figure 1D). We also observed a significant decrease in TEWL for Moisturizers C and L compared with their controls, respectively, but not for Moisturizer K without being calibrated by skin hydration (Figure S4), which might have been caused by skin hydration boosted with higher variability and water evaporation.

All moisturizers resulted in a significant decrease in mean K values (a lower K value indicated a greater proportion of light being scattered away from the skin’s surface, implying increased skin translucency [23]) at 4 W, 8 W, and 8 W + 3 D versus the baseline, except Moisturizer L at 4 W (Figure 1E). Moisturizer K significantly decreased the K value at 4 W compared to that at 1 W, and at 8 W + 3 D compared to that at 8 W, indicating that its benefits lasted even after usage was stopped. Furthermore, Moisturizer L showed significantly less improvement than Moisturizer K at 4 W and 8 W + 3 D (Figure 1F).

No adverse events were reported in the study, and all investigational moisturizers were well-tolerated.

### 3.2. Skin Microbiome Composition

Pooled samples from 97 of 110 participants were used for the analysis. This provided 10,588,824 bacterial 16S ribosomal RNA (rRNA) gene reads of analyzable quality, resulting in 582 bio-samples clustered into 22,239 operational taxonomic units (OTUs) based on a 97% similarity cut-off value. Forty-two bacterial phyla were detected and 1175 bacterial genera were identified, of which 23 had a relative abundance > 0.5%. More specific bacterial phylogenetic analyses were conducted to determine the *Staphylococcus* and *Streptococcus* (abbreviated as *Sta.* and *Str.*, respectively) species or species groups present in the samples based on the amplified V1–V2 regions of the 16S rRNA gene (see Supplementary Materials for a detailed methodology). *Sta. epidermidis* (40.62%), *Sta. capitis* group (29.38%), and *Sta. hominis* (16.05%) were the most abundant species, constituting 86.05% of total staphylococcal sequences. The *Sta. aureus* group, including the highly virulent *Sta. aureus*, which produces coagulase and is abundant in individuals with skin disease, composes a small proportion of the staphylococcal population (1.57%) (Table S2, Figure S5). Among the streptococcal sequences, the *Str. pseudopneumoniae* group (37.94%) was the most abundant, followed by *Str. salivarius* (14.80%), *Str. timonensis* (10.94%), and *Str. sanguinis* (9.33%) (Table S3, Figure S6).

For the fungal community analysis, 8,881,926 analyzable sequences were gathered, forming 4444 OTUs from 582 bio-samples. The fungal dataset primarily comprised the phyla Ascomycota (35.38%) and Basidiomycota (64.26%). Furthermore, 225 distinct and known genera were identified, of which 12 had >0.5% abundance. The most abundant Ascomycota genera were *Alternaria* (13.45%), *Cladosporium* (5.91%), and *Candida* (2.37%). Among Basidiomycota, the most abundant genus was *Malassezia* (56.10%).

### 3.3. Factors Affecting Microbiome Composition and Diversity

The results from a permutational multivariate analysis of variance (PERMANOVA) analysis suggest that, other than individual host factors, lifestyle is a principal factor contributing to bacterial community structure variation (Table 2). This may potentially be attributed to differing exposure levels to environmental factors (i.e., solar radiation, humidity, temperature) between the lifestyle groups. In a further analysis, the 23 most abundant bacterial genera were selected to construct a hierarchical clustering heatmap based on 40 groups combined by different skin sites, sampling times, and occupations (Figure S7), with an occupational clustering pattern revealing that office workers clustered separately from individuals with other jobs. Consistent with the previous studies suggesting that the microbial community composition was stable for up to 2 years [24,25], we found that the sampling time did not have a significant impact on bacterial community composition. However, the skin mycobiome was affected to a greater degree by the site and sampling time than by an environment-dependent lifestyle. Skin sites had a larger effect size on the mycobiome community structure, and sampling time had greater impact on mycobiome community membership (Table 2), indicating that the adult skin mycobiome was less stable over time than previously considered.

Bacterial and fungal alpha diversity indices (Chao 1, Shannon, Observed OTUs, and PD whole tree) at different sites at the baseline are provided in Table S4. The richness estimator (assessed by Chao 1 value) was remained stable between the forehead and upper chest, while other diversity indices, such as the Shannon and PD values, exhibited obvious differences in skin-site effects, proving that Chao 1 was a more suitable indicator to study the effects of the moisturizers on skin microbiome diversity by comparing two skin sites. In the presence of environmental aggressors, there were no temporal variations in the bacterial or fungal community structures of the untreated upper chest samples (Table S5), and the Shannon indices of bacteria on the participants’ upper chest skin samples appeared stable over time (Table S6). However, a decrease in Chao 1 value was observed for both bacteria and fungi (Table S6), suggesting that Chao 1 was a sensitive indicator to study the environmental impact and interventional effects.

### 3.4. Impact of Investigational Moisturizers on the Skin Microbiome

**Figure 2:**
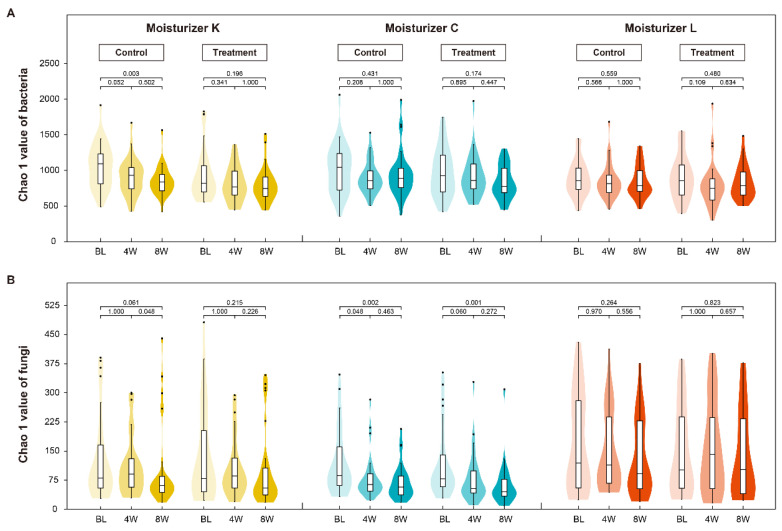
Bacterial (**A**) and fungal (**B**) diversity index Chao 1 values at different sampling times in moisturizers treatment and in untreated control cohorts. Abbreviations: BL, baseline; 4 W, 4 weeks; 8 W, 8 weeks. Significant differences of Chao 1 values between sampling times are exhibited as *p* < 0.05, which are derived from the Kruskal–Wallis test.

Beta diversities of 194 treated forehead samples from 97 participants over two sampling time points (4 and 8 W) were taken. The investigational moisturizers presented obvious effects on the skin’s bacterial community structure (Table 2). While bacterial (BL vs. 8 W: *p* = 0.003) and fungal Chao 1 (4 W vs. 8 W: *p* = 0.048) indices in the untreated samples displayed decreasing richness over time, forehead samples treated with Moisturizer K demonstrated maintenance against the decline in both bacterial (BL vs. 8 W: *p* = 0.196) and fungal Chao 1 values (4 W vs. 8 W: *p* = 0.226) at 8 weeks (Figure 2). Moisturizer C treatment demonstrated the potential to maintain the Chao 1 values of fungi during the first 4 weeks (BL vs. 4 W: *p* = 0.060), as opposed to the declining richness observed in the untreated samples (BL vs. 4 W: *p* = 0.048); however, this effect was less significant as the *p*-value was close to the threshold of 0.05. As no obvious variation tendency was observed in the Moisturizer C bacterial Chao 1 group and both the Moisturizer L bacterial and fungal Chao 1 cohorts, their maintenance effects were not evaluated well using Chao 1 indices, with the result not being significant.

**Figure 3:**
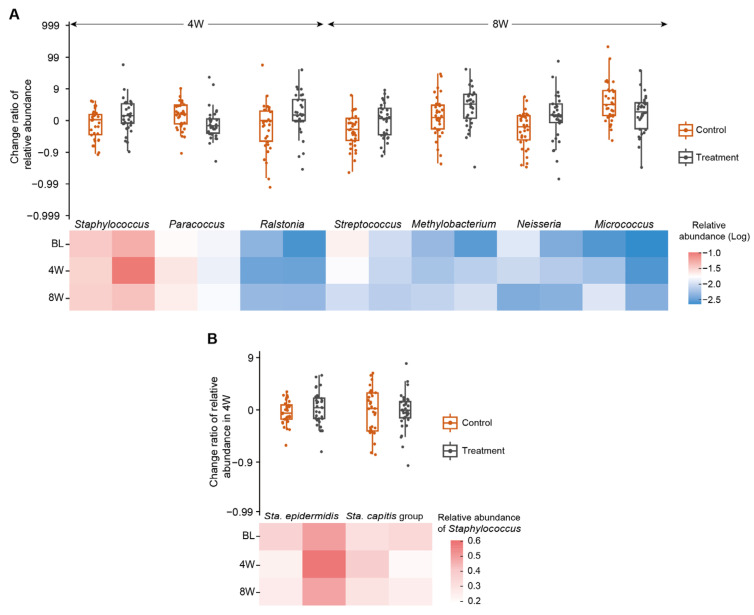
Change ratio of the relative abundance of different taxa among untreated and Moisturizer K (water gel with yeast extract)-treated samples. (**A**) Change ratio of bacterial genera relative abundance shows significant differences at 4 W and 8 W after using Moisturizer K. (**B**) Change ratio of the relative abundance of the genus *Staphylococcus* shows significant differences between moisturizer K treatment and untreated samples at 4 W. The change ratio is calculated as follows: (4 W or 8 W relative abundance—baseline relative abundance)/baseline relative abundance. The minimum value of all non-zero data is selected if the baseline relative abundance value is zero. Abbreviations: 4 W, 4 weeks; 8 W, 8 weeks; BL, baseline; Sta., *Staphylococcus.* Wilcoxon and Kruskal–Wallis tests are performed for difference analysis.

Given the observed spatial and temporal stabilities in the bacterial community (Table 2), the change ratios of the relative abundance of different taxa between the untreated upper chest and forehead samples treated with each investigational moisturizer were used further in the study to minimize the effect of different body sites. The change ratio of bacterial relative abundance varied markedly in the presence of Moisturizer K, with bacteria, such as *Staphylococcus* (*p* = 0.008) and *Ralstonia* (*p* = 0.026), increasing at 4 W, while *Paracoccus* (*p* = 0.028) decreased (Figure 3A, Table S7). Although *Staphylococcus* and *Ralstonia* possessed higher average relative abundances, with *Paracoccus* exhibiting a lower average relative abundance with Moisturizer K at 8 W, their relative abundance change ratios were no longer significant (*p* > 0.050). The change ratio of the relative abundance of *Ralstonia* continued to increase in the presence of Moisturizer K, while the change ratios of *Staphylococcus* and *Paracoccus* showed contrasting results compared with those obtained at 4 W (Table S7), indicating that Moisturizer K treatment was more effective on the aforementioned bacteria at 4 W. At 8 W, the change ratios of the relative abundances of *Streptococcus*, *Methylobacterium* and *Neisseria* showed a significant increase, while *Micrococcus* exhibited a trend towards inhibition (Figure 3A). Furthermore, *Staphylococcus* and *Streptococcus* were classified as species or species groups based on the amplified V1–V2 regions of the 16S rRNA gene (Figures S5 and S6), and the change ratio of different species (groups) showed that Moisturizer K increased the relative abundance of *Sta. epidermidis* and decreased the relative abundance of species in the *Sta. capitis* group (Figure 3B, Table S8). However, compared to Moisturizer K, Moisturizers C and L had no significant effects on the relative abundance of *Staphylococcus* species (Tables S9 and S10). The change ratio of the relative abundance of *Enhydrobacter* was significantly lower in forehead samples treated with Moisturizer C than in the untreated upper chest samples in the first 4 W, while *Finegoldia* was obviously higher in the forehead samples at 8 W (Figure S8A). A decline in the relative abundances of *Streptococcus* and *Rhizobium* was observed with the use of Moisturizer L in the first 4 W, with increasing and decreasing relative abundances of *Lactobacillus* and *Lysobacter* at 8 W, respectively (Figure S8B). Other species did not show directly significant variations after moisturizer usage.

### 3.5. Ex Vivo Chamber Stimulus Analysis

**Figure 4:**
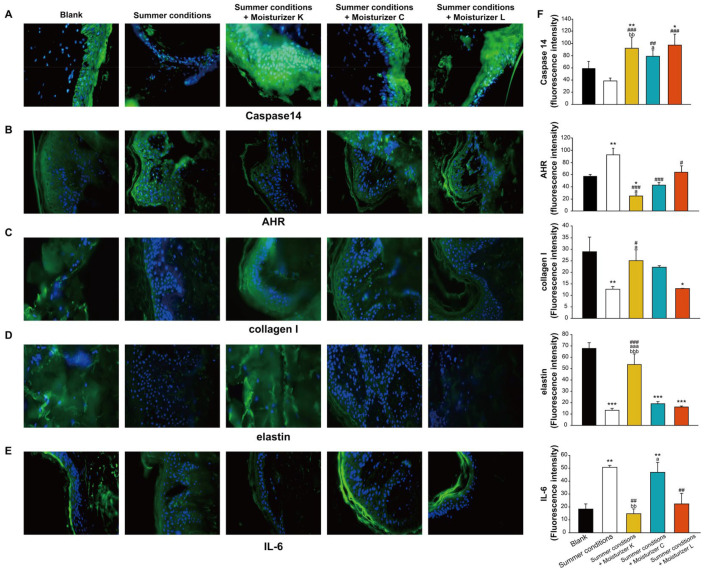
Images of skin explants immunostained for caspase 14 (**A**), AHR receptor (**B**), collagen I (**C**), elastin (**D**), and IL-6 (**E**), and the histograms of their levels (**F**) following simulated summer environment conditions in ex vivo skin samples after the application of the investigated moisturizers. Abbreviations: AHR, aryl hydrocarbon receptor; IL-6, interleukin-6; Moisturizers K, water gel with yeast extract; C, water gel; L, extra dry emulsion. *** *p* < 0.001, ** *p* < 0.01, * *p* < 0.05 versus blank; ### *p* < 0.001, ## *p* < 0.01, # *p* < 0.05 versus summer conditions; aaa *p* < 0.001, a *p* < 0.05 versus summer conditions + Moisturizer L; bbb *p* < 0.001, bb *p* < 0.01 versus summer conditions + Moisturizer C. *p*-values are derived from ANOVA.

An ex vivo study was performed to investigate the protective effects of the moisturizers on human skin biopsies after exposure to simulated environmental aggressors, including urban air pollution and seasonal weather conditions. After one hour of exposure to the simulated environment, skin biopsies were taken and assessed for biomarkers. Caspase 14, a marker of skin barrier function (green fluorescence; Figure 4A), was significantly increased in all moisturizer groups (Moisturizer K: 135%; Moisturizer C: 102%; Moisturizer L: 149%), in comparison with the untreated area, indicating that all investigational moisturizers upregulated caspase-14 expression.

Moisturizer-treated explants also showed significantly lower levels of AHR (green fluorescence; Figure 4B) than those in the untreated areas (ranging from −31% to −72%), suggesting that moisturizers may inhibit AHR activation and potentially play a role in protecting skin samples from environmental stress. Moisturizer K-treated explants showed a significantly lower decrease in collagen I and elastin (green fluorescence; Figure 4C,D) than that in the untreated areas (collagen I: +56%; elastin: +304%), suggesting that moisturizer K could protect against environmental stress-induced reductions in collagen I and elastin. Moreover, Moisturizers K- and L-treated explants also showed significantly lower levels of IL-6 (green fluorescence; Figure 4E) than those in the untreated areas (Moisturizer K: −71%; Moisturizer L: −56%), indicating that Moisturizers K and L could protect against environmental stress-induced inflammation responses.

## 4. Discussion

Our study was the first to evaluate the effects of a topical facial moisturizer application in a Chinese female cohort by pioneering the tracking of real-life environmental aggressors as lifestyle-based environmental factors that affect the skin microbiome (including mycobiome). It thus aimed to put forward the potential aim of moisturizers in maintaining the skin barrier function in the presence of environmental aggressors from the aspects of biomarkers, microbiome composition, and diversity. In this study, we evaluated the performance of three moisturizers on skin health, through an 8-week, randomized controlled, and triple-blind clinical trial, including 110 participants with diverse lifestyles, based on environmental aggressor exposure.

All three types of moisturizers were well-tolerated and were found to improve the skin barrier function and surface moisture content within 1 W, which was maintained until at least 3 days after moisturizer usage was stopped at 8 W (Figure 1). The moisturizers also significantly increased caspase-14 activity (Figure 4A) in a skin explant model, which was key for maintaining skin hydration through the degradation of filaggrin to NMFs in the skin [26]. Caspase 14 also contributes to improve the skin barrier function and protection of the skin against several environmental stressors, such as UV-induced photodamage, which is more likely to occur during summer [27]. While all three moisturizers enhanced skin barrier protection and were clinically beneficial, Moisturizer K showed an extended benefit on skin translucency compared with that of Moisturizer L (Figure 1F). Moreover, Moisturizer K could significantly decrease collagen-I and elastin levels, as well as prevent the IL-6 inflammation response, under an environmental stress model (Figure 4C–E). Kiwi-derived yeast extract has been reported to induce endogenous hyaluronic acid synthase expression and is an effective topical treatment for restoring the levels of hyaluronic acid, a key molecule involved in maintaining skin structure and moisture, and it may result in clinical improvements in skin hydration and radiance [28].

As for the skin microbial barrier, microbiome richness (assessed by Chao 1), an important contributor to skin health [29,30], decreased with fluctuations in the city climate and exposure to environmental aggressors during the study (Table S6). Moisturizers with different formulations were found to provide the skin with varying levels of resilience to decreases in microbiome richness. Moisturizer K prevented decreases in the variety of bacterial and fungal species, whereas Moisturizer C marginally maintained fungal richness and showed no obvious effects on bacterial richness, and Moisturizer L had no significant impact on bacterial and fungal richness (Figure 2). Moisturizer K was formulated as a topical thin oil-in-water emulsion containing a kiwi-derived yeast extract [17,28], in contrast to Moisturizer C (a thin oil-in-water emulsion without yeast extract). This explains how Moisturizer K prevented decreases in the variety of bacterial and fungal species, whereas Moisturizer C marginally maintained fungal richness and showed no obvious effects on bacterial richness. Moisturizer L (a thick oil-in-water emulsion with beeswax-led high viscosity) had no significant impact on bacterial and fungal richness (Figure 2). Furthermore, the formulation chassis of Moisturizer K and that of Moisturizer C was in line with the seasonal sensory preference (water gel). The additional postbiotic technology of Moisturizer K may facilitate greater moisturizer bioavailability, enhancing the effect on the skin compared with that of Moisturizer L, a cream with higher viscosity and ingredients that form a water- and air-blocking film that prevents water loss.

In addition to the better maintenance of bacterial and fungal richness, Moisturizer K enabled the potential growth of beneficial bacteria, including *Sta. epidermidis* and *Ralstonia* (Figure 3). A dramatic increase in the relative abundance of *Staphylococcus* was observed by 4 W in Moisturizer K-treated skin compared with that in untreated skin. Other reports also demonstrated that an increase in beneficial commensal microorganisms to a certain degree might be linked to improvements in skin physiology and health [1,18]. Some strains of *Sta. epidermidis* produce glutamyl endopeptidase, an extracellular serine protease, which inhibits biofilm creation by pathogenic *Sta. aureus* [31,32]. Esp-expressing *Sta. epidermidis* also induces keratinocytes to produce antimicrobial peptides through immune cell signaling [2]. Coagulase-negative *Staphylococcus* strains, including *Sta. epidermidis*, produce novel antibiotics and antimicrobial peptides, which reduce *Sta. aureus* colonization [2,33]. Recent findings from a mouse model also showed that *Sta. epidermidis* secreted a sphingomyelinase, which helped increase the ceramide content in the stratum corneum, enhance skin barrier integrity, and reduce skin dehydration [34]. These findings show that the skin microbiome plays an important role in enhancing the skin barrier function and that increases in the relative abundance of skin commensal bacterial, such as *Sta. epidermidis*, after Moisturizer K application are of clinical interest. Although *Sta. epidermidis* is normally regarded as a beneficial skin microbe, its overabundance can harm the skin via the expression of a cysteine protease [35]. Therefore, the specific function and mechanism of *Sta. epidermidis* need further exploration.

**Figure 5:**
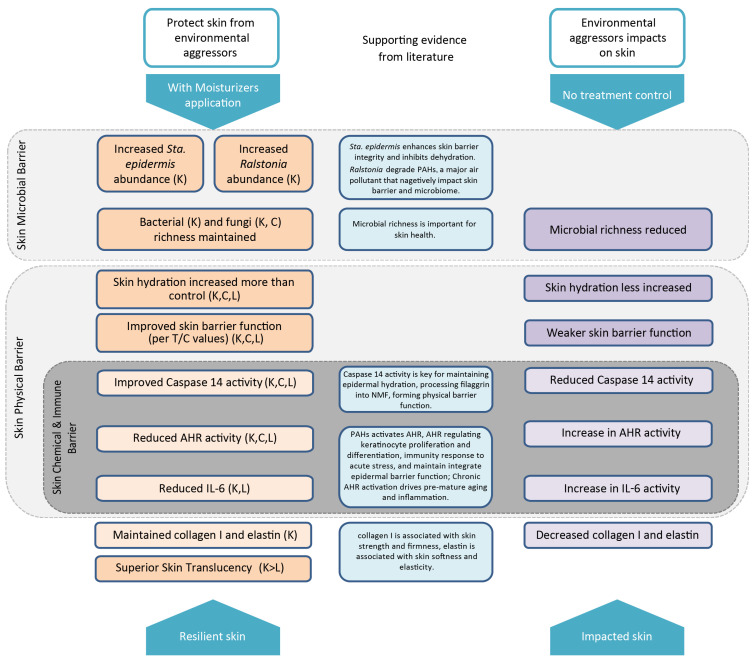
Schematic representation of the impact of environmental aggressors on the skin and the protective action of moisturizers. Abbreviations: K, water gel with yeast extract; C, water gel; L, extra-dry emulsion; AHR, aryl hydrocarbon receptor; PAH, polycyclic aromatic hydrocarbon; *Sta.*, *Staphylococcus*; T/C, ratio of trans-epidermal water loss and Corneometer^®^ value (T/C indicates relative skin barrier function to skin water content). Literature of supporting evidence for each column as follow: [34], [37, 39, 40], [29, 30], [26, 27], [43], and [7].

Our findings of relatively high abundance of *Ralstonia* with Moisturizer K are in line with the previous reports that show an increased relative abundance of *Ralstonia* on facial skin with the use of basic cosmetics [36]. *Ralstonia* is also known to metabolize polycyclic aromatic hydrocarbons (PAHs) [37,38], which are major air pollutants associated with facial skin aging and changes in the skin microbiota with chronic exposure [39,40]. PAHs also lead to the activation of AHR, which is associated with increased expression of epidermal differentiation genes, acceleration of terminal differentiation, and increased stratum corneum thickness [41,42,43]. Although a certain degree of AHR activity is critical for maintaining skin integrity and adapting to acute stressors, chronic AHR activation may drive proinflammatory responses, aging-related gene expression, and melanogenesis [43]. After exposure to simulated urban air pollution, skin explants treated with Moisturizer K exhibited significantly lower levels of AHR than those in the untreated explants, as well as numerically lower AHR levels than those with other moisturizers (Figure 4B). Compared with other moisturizers, Moisturizer K could accelerate the growth of skin commensal and beneficial bacteria, such as *Ralstonia*, which can degrade PAHs, thereby reducing AHR activity and subsequent skin damage. Recent studies have also shown that skin commensal microbes play an important role in regulating AHR skin activity [44]. Thus, a moisturizer that can affect skin microbiome composition and AHR activity may be able to maintain skin barrier function and prevent premature aging (Figure 5).

Overall, Moisturizer K showed healthy-looking benefits concerning skin translucency (Figure 1) with boosted epidermal hydration, biomarkers indicating stronger skin barrier and dermal structure components (Figure 4), exhibited a preferential ability to maintain microbiome richness (Figure 2), and promoted beneficial bacterial species (Figure 3) compared to that with the other two moisturizers. The working model of Moisturizer K was better connected across different levels of skin barrier functions from biomarkers (AHR, caspase 14, IL-6), hydration and barrier function (TEWL), structure components (collagen I and elastin) to microbiome diversity and composition, and it exhibited consistent biomarker responses at each level, suggesting the holistic barrier biological response of Moisturizer K to environmental aggressors (Figure 5).

Moreover, this study had the limitation of having chosen a different body site as the non-moisturizer-used control for the same exposure, and skin explants of only three Caucasian women were chosen to investigate the protective effects of the studied moisturizers on human skin biopsies exposed to environment aggressors. Moreover, the mechanics of how moisturizers affect the skin microbiome and how they protect the skin chemical and immune barriers from environmental aggressors require further investigations and validations.

An ex vivo study was performed to investigate the protective effects of moisturizers on human skin biopsies exposed to environment aggressors, including urban air pollutants and seasonal conditions. Skin explants from three Caucasian women were used.

## 5. Conclusions

Collectively, these findings suggest that, in addition to strengthening the skin barrier, the use of a topical postbiotic moisturizer in the form of a water gel with kiwi-derived yeast extract may improve the skin microbiome composition by increasing microbiome richness and the growth of beneficial bacteria. This may enhance the first layer of the skin barrier defense against the impact of environmental stressors associated with city life and help maintain the resilience of the skin, which in turn serves as a host microenvironment for healthy skin microbiome. Thus, further studies are needed to understand the mechanisms underlying the damage caused by environmental aggressors and the beneficial effects of postbiotic skin moisturizers with bioavailable formulations on the skin microbiome.

## Figures and Tables

**Table 1:** Participant disposition and demographics.

	Moisturizer Type			
	K ^a^	C ^a^	L ^a^	Total
Enrolled participants (n)	40	41	41	122
Completed (drop out) participants for clinical study (n)	37 (3)	35 (6)	38 (3)	110 (12)
Completed (drop out) participants for microbiome study (n)	32 (8)	32 (9)	33 (8)	97 (25)
Age (mean ± SD) ^a^	32.6 ± 5.5	33.2 ± 5.2	32.3 ± 5.6	
Skin type (clinical study; n = 110)				
Normal (n)	5	2	6	13
Dry (n)	24	26	29	79
Oily (n)	0	0	0	0
Combination (n)	8	7	3	18
Sensitive (n)	5	5	1	11
Non-sensitive (n)	32	30	37	99
Lifestyle/occupation (clinical study; n = 110)				
Student (n)	4	2	5	11
Housewife (n)	5	6	7	18
Office worker (n)	23	22	20	65
Outdoor worker (n)	4	5	2	11
Other (n)	1	0	4	5
Lifestyle/occupation (microbiome study; n = 97)				
Student (n)	3	2	4	9
Housewife (n)	5	4	5	14
Office worker (n)	20	21	19	60
Outdoor worker (n)	3	5	2	10
Other (n)	1	0	3	4

^a^ Abbreviations: K, water gel with yeast extract; C, water gel; L, extra-dry emulsion; SD, standard deviation.

**Table 2:** Details of PERMANOVA results from samples grouped by different factors ^a^.

Factor	Bacterial Community				Fungal Community			
	Weighted UniFrac		Unweighted UniFrac		Bray–Curtis		Binary Jaccard	
	F Value	*p*-Value	F Value	*p*-Value	F Value	*p*-Value	F Value	*p*-Value
Site	1.438	0.181	2.574	0.001	52.67	0.001	2.309	0.001
Environment-dependent lifestyle	4.360	0.001	1.542	0.001	1.548	0.037	1.522	0.001
Time	0.976	0.417	1.608	0.001	11.68	0.001	4.127	0.001
Individual	4.665	0.001	1.732	0.001	2.089	0.001	1.921	0.001
Moisturizer ^b^	2.650	0.019	1.192	0.068	1.700	0.044	1.702	0.001

^a^ PERMANOVA, permutational multivariate analysis of variance. ^b^ Moisturizer effects were measured from 194 forehead samples.

## Data Availability

The raw sequencing data amplified in our study were deposited in NODE (the National Omics Data Encyclopedia) under project ID OEP001745 (https://www.biosino.org/node/project/detail/OEP001745, registration on 4 August 2021) and in NCBI GenBank under BioProjects PRJNA757525 (https://www.ncbi.nlm.nih.gov/sra/PRJNA757525, registration on 24 August 2021) and PRJNA757535 (https://www.ncbi.nlm.nih.gov/sra/PRJNA757535, registration on 24 August 2021). ClinicalTrials.gov identifier: NCT03264677.

## Supplementary Materials

The following supporting information can be downloaded at: https://www.mdpi.com/article/10.3390/jcm12186078/s1, Figure S1: Environmental climate reporting during study period; Figure S2: Location of skin evaluations; Figure S3: Set-up of the D-SKIN Cell® apparatus. Figure S4: Comparison of skin trans epidermal water loss between the moisturizers/NT; Figure S5: Maximum likelihood phylogenetic tree based on 16S rRNA V1–V2 region sequences of 53 *Staphylococcus* type strains downloaded from the EzBioCloud 16S database; Figure S6: Maximum likelihood phylogenetic tree based on 16S rRNA V1–V2 region sequences of 110 *Streptococcus* type strains downloaded from the EzBioCloud 16S database; Figure S7: Hierarchical-clustering heat map of 23 major bacterial genera according to 40 groups based on skin sites, sampling times, and occupations; Figure S8: The change ratio of the relative abundance of bacterial genera showed significance at 4 and 8 weeks after using (A) Moisturizer C (water gel) and (B) Moisturizer L (extra dry emulsion); Table S1: Comparison of Moisturizer formulations; Table S2: The proportion (Mean ± SD, %) of major *Staphylococcus* species (groups) in the total staphylococcal reads for each involved skin site at three sampling times; Table S3: The proportion (Mean ± SD) of major *Streptococcus* species (groups) in the total streptococcal reads for each involved skin site at three sampling times; Table S4: Bacterial and fungal alpha diversity indices for different sites at the baseline; Table S5: Details of bacterial and fungal PERMANOVA results for 291 upper chest samples grouped by different factorsa; Table S6: Changing trends in bacterial and fungal alpha diversity indices at different sampling times among 291 upper chest samples; Table S7: The relative abundance of bacterial genera (Mean ± SD) with the significant change ratio (Median, (Q1, Q3)) in the presence of Moisturizer K; Table S8: The relative abundance of major *Staphylococcus* species (Mean ± SD) and their change ratio in the presence of Moisturizer K (Median, (Q1, Q3)); Table S9: The relative abundance of major *Staphylococcus* species (Mean ± SD) and their change ratio in the presence of Moisturizer C (Median, (Q1, Q3)); Table S10: The relative abundance of major *Staphylococcus* species (Mean ± SD) and their change ratio in the presence of Moisturizer L (Median, (Q1, Q3)) [21,45,46,47,48,49,50,51,52,53,54].
