# Supplementary material for: Effects of Investigational Moisturizers on the Skin Barrier and Microbiome following Exposure to Environmental Aggressors: A Randomized Clinical Trial and Ex Vivo Analysis
Source: J Clin Med. 2023 Sep 20;12(18):6078. doi: 10.3390/jcm12186078 (PMC10532330; doi:10.3390/jcm12186078)
Supplement: Supplementary file 1 [file jcm-12-06078-s001.zip › jcm-2553295-supplementary.pdf]

Supplementary material

Dan-Qi Wang [1,†], Xi Li [2,*,†], Ru-Yi Zhang [1], Chao Yuan [3], Bo Yan [2], Philippe Humbert [4] and Zhe-Xue Quan [1,5,*]

[1] Ministry of Education Key Laboratory for Biodiversity Science and Ecological Engineering, Fudan
Microbiome Center, Institute of Biodiversity Science, School of Life Sciences, Fudan University,
Shanghai 200437, China; 18110700020@fudan.edu.cn (D.-Q.W.); 16210700125@fudan.edu.cn (R.-Y.Z.)

[2] Translational Science Asia Pacific, Shanghai Technology and Research Center, Johnson & Johnson (China) Ltd., Shanghai 200245, China; byan7@its.jnj.com

[3] Skin and Cosmetic Research Department, Shanghai Skin Disease Hospital, Shanghai 200443, China; dermayuan@163.com

[4] Department of Dermatology, Clinical Investigation Center, Besancon University Hospital,
25030 Besancon, France; philippehumbert25@gmail.com

[5] IRDR ICoE on Risk Interconnectivity and Governance on Weather/Climate Extremes Impact and Public Health, Fudan University, Shanghai 200437, China

* Correspondence: cli9@kenvue.com (X.L.); quanzx@fudan.edu.cn (Z.-X.Q.); Tel: (8621)-24168018 (X.L.); (8621)-31246665 (Z.X.Q.); Fax: (8621)-64622319 (X.L.); (8621)-31246660 (Z.X.Q.)

† These authors contributed equally to this work.

METHODS

Clinical study details, including inclusion and exclusion criteria, triple-blind design, randomization details, and the qualification and training of dermatologists can be found at: https://clinicaltrials.gov/ct2/show/study/NCT03264677?term=NCT03264677&draw=2&rank=1.

Application of investigational moisturizer

After baseline measurement, the moisturizer regimens were applied to the face by subjects under clinical supervision at the test site according to the randomization form. Subjects first wet their face with warm (30–37°C) tap water, then applied 1 mL (the volume size was demonstrated on-site to guide subject application) of facial cleanser on the palm, lathered and applied it on the whole face, and massaged in a circular motion for 15–20 seconds. They then rinsed their face completely and patted it dry with a lint-free facial tissue (at the site)/towel (at home). Next, the subjects applied 1 mL (the volume size was demonstrated on site to guide subject application) of the facial cream evenly to the whole face and massaged it for complete absorption. Contact with the upper chest area was avoided when applying the moisturizer regimens. Measurements were taken at 2–4 hours and 8 hours after applying the moisturizer regimens. The upper chest area was measured as the no treatment control.

A moisturizer regimen set, which included a facial cleanser and facial cream, was randomly distributed to each subject and applied on the face following the same usage instructions as those during on-site application for 8 weeks. Both the cleanser and cream moisturizer were applied twice a day, in the morning and evening. For the upper chest area, a water wash was used twice a day, in the morning and evening; no other moisturizer was used.

After the 8-week home use of the assigned moisturizer regimen, subjects stopped using the moisturizers for 3 days (regression period). During the regression period, the subjects only used

water to wash their face and upper chest region twice per day, in the morning and evening. Subjects were not allowed to use any other cosmetic moisturizers or topical treatments on the face and upper chest region except for the assigned moisturizers throughout the study period.

DNA extraction, polymerase chain reaction (PCR), and sequencing

Genomic DNA was extracted from the 582 skin surface swab samples using the MoBio PowerSoil® DNA Isolation kit (Qiagen, Valencia, CA, USA) following the manufacturer's instructions, with slight modifications. Briefly, 60 μL of C1 lysis buffer was added to each PowerBead tube (Qiagen) containing swabs and placed in a 65°C water bath for 15 minutes before a beating step was performed on a MOBIO vortex adapter (Qiagen) [1, 2]. The primer 27F (5′-NNNNNNNNNNNN TCAGAGTTTGATCCTGGCTCAG-3′) with 12-nt barcodes, a 2-bp linker (5′-TC-3′), and 338R (5′-TGCTGCCTCCCGTAGGAGT-3′) were used to amplify the 16S ribosomal RNA (rRNA) hypervariable V1–V2 region of bacteria [2]. Each 50-μL reaction comprised 25 μL Ex Taq™ Premix version 2.0 (Takara, Dalian, China), 2 μL of each forward and reverse primer (2.5 μM), 6 μL of template DNA, and 15 μL of ddH_2O. The reaction program comprised an initial denaturation step at 98°C for 5 minutes; followed by 30 cycles of 98°C for 30 seconds, 52°C for 45 seconds, and 72°C for 60 seconds; and a final extension at 72°C for 10 minutes.

Nested polymerase chain reaction (PCR) with dual-barcoded primers was used to target the fungal internal transcribed spacer 1 (ITS1) region; NSA3 (5′-AAACTCTGTCGTGCTGGGGATA-3′) and NLC2 (5′-GAGCTGCATTCCCAAACAACTC-3′) were used for the first round of amplification [3, 4]. Each 50-μL amplicon PCR contained 25 μL Ex Taq™ Premix version 2.0, 2 μL of each forward and reverse primer (2.5 μM), 5 μL of template DNA, 1 μL of bovine serum albumin (20 mg/mL), and 15 μL of ddH_2O. PCR conditions included denaturation at 94°C for 5 minutes;

followed by 30 cycles of 94°C for 30 seconds, 50°C for 45 seconds, and 72°C for 60 seconds; and a final extension at 72°C for 10 minutes. For the second-round PCR, NSI1 (5′-NNNNNNNNNNNN GATTGAATGGCTTAGTGAGG-3′) and 58A2R (5′-NNNNNNNNNNNN CTGCGTTCTTCATCGAT-3′) with 12-nt barcodes were used as primers [4, 5]. The second-round PCR was similar to the first round except that the template was 5 µL of the product from the first round and the number of amplification cycles were decreased from 30 to 25. During amplification for both bacteria and fungi, negative controls were also amplified simultaneously to guarantee no contamination.

PCR-amplified products were purified using the AxyPrep DNA Gel Extraction kit (Axygen, Tewksbury, MA, USA) according to the manufacturer's instructions, and the concentrations were measured using the double-stranded DNA (dsDNA) high-sensitivity (HS) assay (Invitrogen, Carlsbad, CA, USA). Samples were pooled with equal mole concentrations and used for library construction with the LTP Library Preparation kit (KAPA Biosystems, Boston, MA, USA). DNA amplicons were sequenced on an Illumina MiSeq platform (Illumina, San Diego, CA, USA) to generate 2 x 250 paired-end reads.

Sequence analyses of skin microbiomes

Post-sequencing data analysis was mainly performed following the Quantitative Insights Into Microbial Ecology (QIIME) version 1.8 pipeline (http://www.qiime.org) [6]. For bacterial sequencing analysis, all paired-end reads were qualified using FastQC (http://www.bioinformatics.babraham.ac.uk/projectes/fastqc/) and filtered using Sickle (https://github.com/najoshi/sickle) with a minimum Phred score of 20. Subsequently, paired-end reads were merged into one sequence and sequences shorter than 150 bp were removed; the remaining sequences were demultiplexed with 12-nt barcodes and chimeras were checked using USEARCH61 (http://drive5.com/usearch/usearch_docs.html) against the SSU Ref NR (small subunit rRNA gene reference non-redundant) 132 database (https://www.arb-

silva.de/projects/ssu-ref-nr/) [7]. After chimeras were removed, samples with a depth of fewer than 5000 reads were excluded, and the remaining sequences were clustered into operational taxonomic units (OTUs) based on a 97% identity threshold using the QIIME script "pick_open_reference_otus"; taxonomy information was then assigned using Ribosomal Database Project classifiers [8]. To perform taxonomic classifications at the species level for *Staphylococcus* and *Streptococcus*, sequences related to the genus *Staphylococcus* and *Streptococcus* were extracted for more sophisticated processing using the QIIME script "pick_de_novo_otus" function based on the 99% identity threshold. Custom species-level reference datasets were established using 53 and 110 complete 16S rRNA sequences of *Staphylococcus*- and *Streptococcus*-type strains downloaded from the EzBioCloud 16S database (https://www.ezbiocloud.net/resources/16s_download) [9], respectively.

The quality of fungal paired-end reads was also verified using FastQC and their orientation was adjusted based on the primer sequences. For both Read 1 and Read 2 sequences, the first 200 nt were trimmed and retained as the Phred scores of the last 50 nt bases of the reads were mostly lower than 20. After quality control, the chimeras of the complete dataset were identified against the UNITE database [10] using USEARCH61 and sequences were assembled by placing the reverse complement sequences of Read 2 in front of Read 1, further assigned to samples according to their barcodes. To describe community structures within the cohort more accurately and enhance representation, samples with less than 4000 reads were discarded. Sequences were clustered to OTUs by 95% identity threshold using the QIIME script "pick_open_reference_otus", and taxonomy was assigned to sequences with the RDP Classifier. Based on the taxonomic assignment results, all reads belonging to the order Malasseziales were separated and re-assigned to OTUs by a 99% identity threshold. According to the newly generated table, 92 OTUs accounted for 92.85% of the total; Malasseziales sequences whose proportions were greater than 0.02% were chosen to construct a maximum-

likelihood phylogenetic tree. A K2+G model in Mega X [11] and taxonomy information were ensured by BLASTN searches against standard National Center for Biotechnology Information (NCBI) databases. OTUs with an abundance of less than 0.02% were classified as ‘others’.

REFERENCES

1. Ying S, Zeng DN, Chi L, Tan Y, Galzote C, Cardona C, Lax S, Gilbert J, and Quan ZX. The Influence of Age and Gender on Skin-Associated Microbial Communities in Urban and Rural Human Populations. *PLoS One.* **2015**, *10*, e0141842. https://doi.org/10.1371/journal.pone.0141842.

2. Zhu T, Liu X, Kong FQ, Duan YY, Yee AL, Kim M, Galzote C, Gilbert JA, and Quan ZX. Age and Mothers: Potent Influences of Children's Skin Microbiota. *J Invest Dermatol.* **2019**, *132*, 2497-2505. https://doi.org/10.1016/j.jid.2019.05.018.

3. Toju H, Tanabe A S, Yamamoto S, and Sato H. High-coverage ITS primers for the DNA-based identification of ascomycetes and basidiomycetes in environmental samples. *PLoS One.* **2012**, *7*, e40863. https://doi.org/10.1371/journal.pone.0040863.

4. Zhu T, Duan YY, Kong FQ, Galzote C, and Quan ZX. Dynamics of Skin Mycobiome in Infants. *Front Microbiol.* **2020**, *11*, 1790. https://doi.org/10.3389/fmicb.2020.01790.

5. Hamady M, Walker JJ, Harris JK, Gold NJ, and Knight R. Error-correcting barcoded primers for pyrosequencing hundreds of samples in multiplex. *Nat methods.* **2008**, *5*, 235-237. https://doi.org/10.1038/nmeth.1184.

6. Caporaso JG, Kuczynski J, Stombaugh J, Bittinger K, Bushman FD, Costello EK, Fierer, N, Peña AG, Goodrich JK, Gordon JI, Huttley GA, Kelley ST, Knights D, Koenig JE, Ley RE, Lozupone CA, McDonald D, Muegge BD, Pirrung M, Reeder J, Sevinsky JR, Turnbaugh PJ, Walters WA, Widmann J, Yatsunenko T, Zaneveld J, and Knight R. QIIME allows analysis of high-throughput community sequencing data. *Nat methods.* **2010**, *7*, 335-336. https://doi.org/10.1038/nmeth.f.303.

7. Quast C, Pruesse E, Yilmaz P, Gerken J, Schweer T, Yarza P, Peplies J, and Glockner F O. The SILVA ribosomal RNA gene database project: improved data processing and web-based tools. *Nucleic Acids Res.* **2013**, *41*, D590-596. https://doi.org/10.1093/nar/gks1219.

8. Wang Q, Garrity MG, Tiedje JM, and Cole JR. Naive Bayesian classifier for rapid assignment of rRNA sequences into the new bacterial taxonomy. *Appl Environ Microbiol.* **2007**, *73*, 5261-5267. https://doi.org/10.1128/AEM.00062-07.

9. Yoon SH, Ha SM, Kwon S, Lim J, Kim Y, Seo H, and Chun J. Introducing EzBioCloud: a taxonomically united database of 16S rRNA gene sequences and whole-genome assemblies. *Int J Syst Evol Microbiol.* **2017**, *67*, 1613-1617. https://doi.org/10.1099/ijsem.0.001755.

10. Nilsson RH, Larsson KH, Taylor AFS, Bengtsson-Palme J, Jeppesen TS, Schigel D, Kennedy P, Picard K, Glockner FO, Tedersoo L, Saar I, Koljalg U, and Abarenkov K. The UNITE database for molecular identification of fungi: handling dark taxa and parallel taxonomic classifications. *Nucleic Acids Res.* **2019**, *47*, D259-D264. https://doi.org/10.1093/nar/gky1022.

11. Kumar S, Stecher G, Li M, Knyaz C, and Tamura K. MEGA X: Molecular Evolutionary Genetics Analysis across Computing Platforms. *Mol Biol Evol.* **2018**, *35*, 1547-1549. https://doi.org/10.1093/molbev/msy096.

Supplemental Tables

Table S1. Comparison of Moisturizer formulations

	Moisturizer K	Moisturizer C	Moisturizer L
Product name	Neutrogena Hydro Boost Kiwi Water Gel (water gel with yeast extract)	Neutrogena Hydro Boost Water Gel (water gel)	Neutrogena Hydro Boost Extra Dry Emulsion (extra dry emulsion)
Formula composition, %			
Formula base (oil in water emulsion)	95.0	100.0	99.5
Emulsifier	1	1	2
Humectant	3	3	6
Preservatives	1	1	1
Synthetic beeswax	0	0	0.5
Yeast extract	5.0	0	0
pH	5.5	5.5	5.5
Viscosity, cpsa	33,000	33,000	50,000

[a]Abbreviation: cps, centipoise.

Table S2. The proportion (Mean ± SD, %) of major *Staphylococcus* species (groups) in the total staphylococcal reads for each involved skin site at three sampling times

***Staphylococcus* spp.**	**Phylogenetic cluster in this study**	**Baseline**		**Week 4**		**Week 8**	
		**Upper chest**	**Forehead**	**Upper chest**	**Forehead**	**Upper chest**	**Forehead**
Staphylococcus epidermidis	*Sta. epidermidis*	36.2 ± 17.0	50.1 ± 24.6	31.3 ± 16.8	51.1 ± 24.3	30.6 ± 17.6	45.9 ± 22.6
Staphylococcus caprae<br>*Staphylococcus capitis*<br>*Staphylococcus saccharolyticus*	*Sta. capitis* group	31.5 ± 18.9	31.2 ± 23.3	31.8 ± 21.5	27.5 ± 21.8	28.8 ± 22.4	26.7 ± 20.9
Staphylococcus hominis	*Sta. hominis*	18.7 ± 16.2	8.25 ± 9.77	19.8 ± 16.7	8.37 ± 9.14	24.9 ± 19.5	14.8 ± 13.6
Staphylococcus warneri	*Sta. warneri*	3.05 ± 3.79	2.61 ± 3.79	4.62 ± 7.02	4.71 ± 6.81	4.15 ± 5.83	3.55 ± 7.39
Staphylococcus haemolyticus	*Sta. haemolyticus*	2.61 ± 3.32	1.31 ± 1.91	3.07 ± 5.23	1.70 ± 2.95	3.16 ± 6.08	1.73 ± 3.20
Staphylococcus cohnii<br>*Staphylococcus arlettae*	*Sta. cohnii* group	2.69 ± 3.71	1.80 ± 3.36	2.33 ± 3.71	1.47 ± 2.97	2.28 ± 3.33	2.39 ± 4.14
Staphylococcus aureus<br>*Staphylococcus schweitzeri*<br>*Staphylococcus argenteus*	*Sta. aureus* group	1.48 ± 7.56	2.07 ± 8.19	2.10 ± 6.02	1.51 ± 3.22	1.18 ± 4.12	1.06 ± 2.82
Staphylococcus edaphicus<br>*Staphylococcus saprophyticus*<br>*Staphylococcus pseudoxylosus*	*Sta. edaphicus* group	0.71 ± 2.72	0.48 ± 1.36	1.69 ± 4.91	1.49 ± 6.16	1.68 ± 4.71	1.00 ± 2.82

Table S3. The proportion (Mean ± SD) of major *Streptococcus* species (groups) in the total streptococcal reads for each involved skin site at three sampling times

***Streptococcus* spp.**	**Phylogenetic cluster in this study**	**Baseline**		**Week 4**		**Week 8**	
		**Upper chest**	**Forehead**	**Upper chest**	**Forehead**	**Upper chest**	**Forehead**
Streptococcus pneumoniae<br>*Streptococcus mitis*<br>*Streptococcus pseudopneumoniae*<br>*Streptococcus oralis*<br>*Streptococcus chosunense*	*Str. pseudopneumoniae* Group	38.7 ± 25.3	39.2 ± 28.5	36.1 ± 31.1	35.6 ± 28.4	38.7 ± 29.4	38.5 ± 33.2
Streptococcus salivarius<br>*Streptococcus thermophilus*<br>*Streptococcus vestibularis*	*Str. salivarius* Group	14.1 ± 17.1	16.5 ± 19.2	12.5 ± 17.7	15.7 ± 17.4	15.7 ± 17.8	15.8 ± 18.6
Streptococcus timonensis<br>*Streptococcus cristatus*	*Str. timonensis* Group	10.9 ± 13.8	10.3 ± 11.7	12.3 ± 20.0	11.5 ± 14.9	11.2 ± 15.3	8.93 ± 12.9
Streptococcus sanguinis	*Str. sanguinis*	9.69 ± 7.81	8.59 ± 9.45	9.97 ± 9.97	9.47 ± 9.32	8.93 ± 9.72	9.45 ± 10.8
Streptococcus infantis	*Str. infantis*	3.16 ± 5.24	2.68 ± 4.17	3.99 ± 5.79	2.95 ± 4.42	3.24 ± 6.40	2.77 ± 5.45
Streptococcus peroris	*Str. peroris*	3.14 ± 5.27	2.64 ± 4.48	3.87 ± 7.94	2.89 ± 5.42	2.04 ± 3.95	2.23 ± 4.27
Streptococcus parasanguinis	*Str. parasanguinis*	2.92 ± 5.84	2.29 ± 5.04	2.35 ± 4.77	3.63 ± 6.45	2.55 ± 5.46	2.88 ± 4.96
Streptococcus dentisani	*Str. dentisani*	2.45 ± 5.10	1.84 ± 4.23	1.51 ± 5.54	1.31 ± 3.77	1.31 ± 3.12	1.78 ± 4.09
Streptococcus gordonii	*Str. gordonii*	1.60 ± 2.99	3.13 ± 5.10	1.94 ± 3.53	2.96 ± 6.00	2.21 ± 4.41	2.42 ± 4.78
Streptococcus anginosus	*Str. anginosus*	1.79 ± 4.36	1.14 ± 4.03	3.20 ± 9.68	0.74 ± 2.17	1.53 ± 4.83	2.02 ± 5.42
Streptococcus lutetiensis<br>*Streptococcus infantarius*<br>*Streptococcus equinus*	*Str. infantarius* Group	1.31 ± 4.70	0.57 ± 2.64	0.93 ± 4.59	1.28 ± 4.62	1.41 ± 4.49	1.90 ± 6.46

Table S4. Bacterial and fungal alpha diversity indices for different sites at the baseline.

Type	Alpha diversity indices	Site (mean ± SD)[a]		*P*-value (Wilcoxon)
		Upper chest	Forehead	
Bacterial	Chao 1	986 ± 308	935 ± 332	0.279[b]
	Shannon	5.72 ± 1.17	5.03 ± 1.32	<0.001
	PD whole tree[a]	9.89 ± 2.78	8.67 ± 2.51	<0.001
	Observed OTUs[a]	552 ± 163	493 ± 154	0.006
Fungi	Chao 1	139 ± 108	132 ± 107	0.141[b]
	Shannon	2.63 ± 0.81	2.29 ± 0.97	0.004
	Observed OTUs[a]	72.7 ± 48.4	70.1 ± 50.2	0.372

[a]Abbreviations: OTU, operational taxonomic unit; PD, phylogenetic diversity; SD, standard deviation.

[b]Chao 1 maintained stable cross sites, which is a better indicator of skin microbiome than other diversity indices.

Table S5. Details of bacterial and fungal PERMANOVA results for 291 upper chest samples grouped by different factors[a]

Details (number of samples)	Time	Occupation	Time	Occupation
Bacteria	Weighted UniFrac distance		Unweighted UniFrac distance	
F value	0.932	1.243	0.915	1.034
P-value	0.369	0.437	0.836	0.265
Fungi	Bray-Curtis distance		Binary Jaccard distance	
F value	0.473	2.233	0.866	1.210
P-value	0.994	0.001	0.953	0.008

[a]Abbreviation: PERMANOVA, permutational multivariate analysis of variance.

Table S6. Changing trends in bacterial and fungal alpha diversity indices at different sampling times among 291 upper chest samples

Site	Type	Alpha diversity indices	Mean ± SD[a]			*P* (Kruskal-Wallis)	*P* (Dunn's test) Bonferroni correction		
			BL[a]	4W[a]	8W[a]		BL vs. 4W	BL vs. 8W	4W vs. 8W
Upper chest (n = 97)	Bacterial	Chao 1	986 ± 308	882 ± 238	880 ± 258	**0.010**	**0.026**	**0.011**	1.000
		Shannon	5.72 ± 1.17	5.87 ± 1.02	5.76 ± 1.26	0.630	0.519	0.771	1.000
		PD whole tree[a]	9.89 ± 2.78	9.11 ± 2.34	9.51 ± 2.43	0.120	0.063	0.335	0.624
		No. of OTUs[a]	552 ± 163	514 ± 118	521 ± 141	0.180	0.172	0.163	1.000
	Fungi	Chao 1	138 ± 108	113 ± 83.9	99.4 ± 88.1	**<0.001**	0.278	**0.001**	0.052
		Shannon	2.63 ± 0.81	2.80 ± 0.70	2.44 ± 0.80	**<0.001**	0.113	0.130	**0.001**
		No. of OTUs[a]	72.7 ± 48.4	64.0 ± 40.3	55.5 ± 41.7	**<0.001**	0.676	**0.001**	**0.014**

[a]Abbreviation: 4W, 4 weeks; 8W, 8 weeks; BL, baseline; OTU, operational taxonomic unit; PD, phylogenetic diversity; SD, standard deviation.

Table S7. The relative abundance of bacterial genera (Mean ± SD) with the significant change ratio (Median, (Q1, Q3)) in the presence of Moisturizer K

	**Baseline**		**Week 4**		**change ratio for 4W**			**Week 8**		**change ratio for 8W**		
	**Upper chest**	**Forehead**	**Upper chest**	**Forehead**	**Upper chest**	**Forehead**	***P* value**	**Upper chest**	**Forehead**	**Upper chest**	**Forehead**	***P* value**
Staphylococcus	3.73% ± 3.44%	5.24% ± 6.21%	3.21% ± 2.80%	10.8% ± 17.2%	0.08 (-0.66, 0.60)	0.46 (-0.16, 2.52)	**0.008**	3.36% ± 3.50%	4.06% ± 3.79%	-0.22 (-0.56, 0.58)	-0.06 (-0.55, 0.90)	0.993
Paracoccus	1.92% ± 3.64%	1.52% ± 2.34%	2.50% ± 2.51%	1.43% ± 1.91%	0.64 (-0.19, 2.08)	-0.32 (-0.60, 0.17)	**0.028**	2.28% ± 3.03%	1.69% ± 2.08%	0.45 (-0.35, 1.18)	0.45 (-0.16, 1.01)	0.919
Ralstonia	0.48% ± 0.86%	0.27% ± 0.64%	0.36% ± 0.96%	0.35% ± 0.79%	0.00 (-0.79, 1.04)	0.49 (0.00, 3.63)	**0.026**	0.53% ± 0.90%	0.55% ± 1.12%	0.21 (-0.02, 1.20)	0.86 (0.00, 3.51)	0.381
Streptococcus	2.19% ± 2.74%	1.01% ± 1.49%	1.73% ± 2.13%	0.91% ± 1.22%	-0.37 (-0.73, 0.53)	-0.18 (-0.65, 1.19)	0.432	1.02% ± 1.76%	0.80% ± 1.25%	-0.54 (-0.81, 0.03)	0.03 (-0.69, 1.11)	**0.021**
Methylobacterium	0.54% ± 1.06%	0.30% ± 0.52%	0.64% ± 0.91%	0.79% ± 1.76%	0.31 (-0.33, 1.12)	0.64 (-0.49, 1.66)	0.210	0.86% ± 1.76%	1.07% ± 1.90%	0.15 (-0.54, 1.69)	1.86 (0.11, 4.78)	**0.024**
Neisseria	1.25% ± 2.99%	0.41% ± 0.99%	0.97% ± 3.02%	0.75% ± 2.60%	-0.40 (-0.70, 0.70)	0.29 (-0.49, 1.66)	0.350	0.41% ± 1.05%	0.46% ± 1.35%	-0.46 (-0.80, 0.19)	0.32 (-0.37, 1.90)	**0.005**
Micrococcus	0.28% ± 0.41%	0.23% ± 0.41%	0.62% ± 1.25%	0.28% ± 0.45%	0.44 (-0.43, 2.64)	0.50 (-0.41, 1.75)	0.500	1.21% ± 2.26%	0.44% ± 0.76%	1.83 (0.29, 6.72)	0.84 (-0.17, 2.12)	**0.050**

Table S8. The relative abundance of major *Staphylococcus* species (Mean ± SD) and their change ratio in the presence of Moisturizer K (Median, (Q1, Q3))

***Staphylococcus* spp.**	**Phylogenetic cluster in this study**	**Baseline**		**Week 4**		**change ratio for 4W**			**Week 8**		**change ratio for 8W**		
		**Upper chest**	**Forehead**	**Upper chest**	**Forehead**	**Upper chest**	**Forehead**	***P* value**	**Upper chest**	**Forehead**	**Upper chest**	**Forehead**	***P* value**
Staphylococcus epidermidis	*Sta. epidermidis*	36.1% ± 17.6%	49.7% ± 25.4%	25.4% ± 14.0%	57.6% ± 28.7%	-0.38 (-0.49, -0.07)	0.07 (-0.20, 0.55)	**0.001**	27.3% ± 15.8%	48.2% ± 24.1%	-0.15 (-0.67, 0.32)	-0.03 (-0.24, 0.32)	0.270
Staphylococcus caprae<br>*Staphylococcus capitis*<br>*Staphylococcus saccharolyticus*	*Sta. capitis* Group	32.2% ± 22.6%	34.5% ± 23.2%	38.6% ± 25.2%	22.6% ± 23.6%	0.12 (-0.13, 0.63)	-0.49 (-0.71, -0.04)	**0.002**	30.1% ± 25.5%	26.1% ± 23.8%	-0.18 (-0.47, 0.44)	-0.37 (-0.65, 0.00)	0.178
Staphylococcus hominis	*Sta. hominis*	19.5% ± 15.8%	8.2% ± 11.9%	17.7% ± 15.8%	6.91% ± 7.71%	-0.21 (-0.51, 0.40)	-0.15 (-0.76, 1.07)	0.905	27.0% ± 18.5%	14.2% ± 12.8%	0.37 (-0.23, 1.68)	1.43 (0.11, 3.94)	0.102
Staphylococcus warneri	*Sta. warneri*	2.97% ± 2.74%	2.70% ± 3.77%	6.16% ± 7.76%	4.50% ± 5.25%	0.48 (-0.62, 3.80)	0.42(-0.48, 3.60)	0.719	3.70% ± 5.71%	3.04% ± 3.72%	-0.32 (-0.90, 0.92)	-0.10 (-0.76, 1.66)	0.129
Staphylococcus haemolyticus	*Sta. haemolyticus*	2.70% ± 2.62%	1.17% ± 1.52%	3.13% ± 6.27%	1.33% ± 2.04%	-0.20 (-0.85, 2.06)	-0.08 (-0.86, 3.01)	0.905	3.53% ± 5.56%	2.44% ± 4.68%	-0.01 (-0.92, 2.05)	0.00 (-0.68, 8.07)	0.477
Staphylococcus arlettae<br>*Staphylococcus cohnii*	*Sta. cohnii* Group	1.99% ± 2.09%	1.37% ± 1.70%	2.02% ± 3.58%	1.26% ± 2.65%	-0.17 (-0.81, 0.85)	-0.58 (-0.98, 3.10)	0.761	1.95% ± 2.69%	1.68% ± 3.08%	-0.37 (-0.97, 0.62)	0.00 (-0.67, 2.79)	0.903

Staphylococcus schweitzeri<br>*Staphylococcus aureus*<br>*Staphylococcus argenteus*	*Sta. aureus* Group	0.83% ± 2.22%	0.58% ± 1.00%	1.02% ± 3.12%	1.30% ± 3.42%	0.00 (-0.74, 6.17)	0.00 (-0.70, 0.08)	0.134	1.34% ± 4.33%	1.44% ± 3.93%	0.00 (-0.68, 8.95)	0.00 (-0.64, 4.04)	0.846
Staphylococcus edaphicus<br>*Staphylococcus saprophyticus*<br>*Staphylococcus pseudoxylosus*	*Sta. edaphicus* Group	0.59% ± 1.01%	0.34% ± 0.78%	2.34% ± 7.63%	2.29% ± 10.34%	0.10 (0.00, 11.55)	0.00 (-0.08, 3.91)	0.703	1.74% ± 4.88%	0.25% ± 0.47%	0.00 (0.00, 0.76)	0.00 (-0.45, 1.84)	0.801
Staphylococcus pettenkoferi	*Sta. pettenkoferi*	0.93% ± 1.40%	0.35% ± 0.73%	1.70% ± 3.66%	0.81% ± 3.01%	0.00 (-0.07, 6.25)	0.00 (-0.72, 3.35)	0.746	1.11% ± 2.25%	0.97% ± 3.50%	0.00 (-0.92, 6.77)	0.00 (-0.42, 1.56)	0.936

Table S9. The relative abundance of major *Staphylococcus* species (Mean ± SD) and their change ratio in the presence of Moisturizer C (Median, (Q1, Q3))

***Staphylococcus* spp.**	**Phylogenetic cluster in this study**	**Baseline**		**Week 4**		**change ratio for 4W**			**Week 8**		**change ratio for 8W**		
		**Upper chest**	**Forehead**	**Upper chest**	**Forehead**	**Upper chest**	**Forehead**	***P* value**	**Upper chest**	**Forehead**	**Upper chest**	**Forehead**	***P* value**
Staphylococcus epidermidis	*Sta. epidermidis*	36.1% ± 17.5%	50.3% ± 20.2%	32.3% ± 17.2%	46.4% ± 23.0%	0.02 (-0.42, 0.29)	-0.16 (-0.30, 0.22)	0.905	34.9% ± 18.6%	45.7% ± 21.1%	-0.03 (-0.32, 0.68)	-0.02 (-0.39, 0.18)	0.203
Staphylococcus caprae *Staphylococcus capitis* *Staphylococcus saccharolyticus*	*Sta. capitis* Group	31.5% ± 16.4%	29.1% ± 16.2%	32.3% ± 21.6%	32.4% ± 19.2%	-0.12 (-0.49, 0.50)	0.05 (-0.44, 0.75)	0.360	26.5% ± 19.6%	26.7% ± 19.2%	-0.27 (-0.49, 0.02)	-0.09 (-0.50, 0.54)	0.262
Staphylococcus hominis	*Sta. hominis*	19.9% ± 15.3%	9.5% ± 8.1%	22.0% ± 18.0%	9.10% ± 9.27%	0.32 (-0.44, 0.65)	-0.14 (-0.63, 0.94)	0.651	24.7% ± 18.1%	15.6% ± 12.2%	0.18 (-0.22, 1.61)	0.82 (-0.28, 2.92)	0.102
Staphylococcus warneri	*Sta. warneri*	3.33% ± 4.63%	3.68% ± 4.66%	2.61% ± 3.86%	5.35% ± 8.66%	-0.29 (-0.65, 0.87)	0.11 (-0.74, 1.73)	0.875	2.94% ± 3.29%	2.34% ± 3.40%	-0.04 (-0.67, 1.38)	-0.63 (-0.90, 0.20)	0.166
Staphylococcus haemolyticus	*Sta. haemolyticus*	1.88% ± 2.62%	1.50% ± 2.16%	3.45% ± 4.48%	2.06% ± 3.56%	0.45 (-0.72, 17.09)	-0.30 (-0.88, 7.66)	0.421	1.90% ± 4.19%	1.46% ± 2.33%	-0.27 (-0.94, 5.96)	0.00 (-0.70, 1.43)	0.561
Staphylococcus arlettae *Staphylococcus cohnii*	*Sta. cohnii* Group	2.48% ± 3.27%	1.81% ± 2.92%	1.53% ± 2.43%	1.12% ± 2.03%	-0.40 (-0.96, 1.32)	0.01 (-0.76, 1.08)	0.761	2.57% ± 3.78%	3.19% ± 5.49%	-0.06 (-0.96, 1.70)	0.00 (-0.91, 2.53)	0.875

Staphylococcus schweitzeri<br><br>*Staphylococcus aureus*<br><br>*Staphylococcus argenteus*	*Sta. aureus* Group	0.35% ± 0.72%	0.45% ± 1.04%	0.66% ± 1.58%	0.47% ± 0.77%	0.00 (-0.86, 3.07)	0.00 (-0.80, 1.56)	0.818	0.37% ± 0.57%	0.72% ± 2.45%	0.00 (-0.70, 16.4)	0.00 (-0.76, 0.00)	0.119
Staphylococcus edaphicus<br><br>*Staphylococcus saprophyticus*<br><br>*Staphylococcus pseudoxylosus*	*Sta. edaphicus* Group	0.49% ± 0.98%	0.55% ± 1.15%	0.72% ± 1.36%	0.89% ± 1.67%	0.00 (-0.57, 9.85)	0.00 (-0.70, 10.99)	0.589	1.91% ± 6.31%	0.74% ± 1.96%	0.00 (-0.69, 26.6)	0.00 (-0.93, 3.94)	0.074
Staphylococcus pettenkoferi	*Sta. pettenkoferi*	0.68% ± 1.21%	0.81% ± 1.78%	0.67% ± 1.42%	0.56% ± 1.08%	0.00 (-0.55, 0.25)	0.00 (-0.44, 1.78)	0.609	0.66% ± 1.43%	0.23% ± 0.42%	0.00 (-0.63, 4.52)	0.00 (-0.91, 0.00)	0.148

Table S10. The relative abundance of major *Staphylococcus* species (Mean ± SD) and their change ratio in the presence of Moisturizer L (Median, (Q1, Q3))

***Staphylococcus* spp.**	**Phylogenetic cluster in this study**	**Baseline**		**Week 4**		**change ratio for 4W**			**Week 8**		**change ratio for 8W**		
		**Upper chest**	**Forehead**	**Upper chest**	**Forehead**	**Upper chest**	**Forehead**	***P* value**	**Upper chest**	**Forehead**	**Upper chest**	**Forehead**	***P* value**
Staphylococcus epidermidis	*Sta. epidermidis*	36.2% ± 16.8%	48.8% ± 26.9%	35.7% ± 18.1%	48.7% ± 20.0%	0.07 (-0.33, 0.39)	0.04 (-0.33, 0.65)	0.778	29.3% ± 17.9%	42.4% ± 22.5%	-0.16 (-0.54, 0.07)	-0.19 (-0.36, 0.21)	0.502
Staphylococcus caprae<br>*Staphylococcus capitis*<br>*Staphylococcus saccharolyticus*	*Sta. capitis* Group	28.4% ± 14.5%	29.1% ± 23.4%	22.7% ± 11.9%	26.4% ± 18.0%	-0.27 (-0.51, 0.34)	0.05 (-0.57, 0.50)	0.537	26.9% ± 18.4%	27.1% ± 19.4%	-0.06 (-0.44, 0.40)	-0.10 (-0.43, 1.34)	0.339
Staphylococcus hominis	*Sta. hominis*	17.6% ± 18.3%	7.53% ± 9.52%	20.3% ± 17.1%	9.30% ± 10.6%	0.46 (-0.29, 1.08)	0.43 (-0.39, 2.86)	0.321	24.8% ± 22.2%	15.3% ± 16.0%	0.48 (-0.22, 2.44)	1.62 (0.33, 8.91)	0.304
Staphylococcus warneri	*Sta. warneri*	3.07% ± 3.97%	1.69% ± 2.64%	5.35% ± 8.49%	4.66% ± 6.47%	0.26 (-0.58, 7.18)	1.56 (0.19, 9.31)	0.480	5.63% ± 7.36%	5.43% ± 11.8%	0.06 (-0.76, 7.02)	0.25 (-0.57, 9.50)	0.764
Staphylococcus haemolyticus	*Sta. haemolyticus*	3.35% ± 4.36%	1.33% ± 2.10%	2.87% ± 5.12%	1.78% ± 3.17%	-0.57 (-0.94, 1.26)	0.00 (-0.17, 1.63)	0.360	4.25% ± 7.99%	1.32% ± 1.97%	-0.61 (-0.93, 1.15)	0.00 (-0.69, 3.47)	0.992
Staphylococcus arlettae<br>*Staphylococcus cohnii*	*Sta. cohnii* Group	3.66% ± 4.62%	2.29% ± 4.44%	3.44% ± 4.45%	2.13% ± 3.77%	0.00 (-0.74, 1.09)	0.14 (-0.44, 3.85)	0.437	2.35% ± 2.90%	2.44% ± 2.92%	-0.21 (-0.81, 3.37)	1.16 (-0.49, 24.5)	0.711

Staphylococcus schweitzeri<br>*Staphylococcus aureus*<br>*Staphylococcus argenteus*	*Sta. aureus* Group	3.36% ± 12.87%	5.30% ± 13.76%	4.60% ± 9.50%	2.56% ± 4.12%	0.00 (-0.09, 41.3)	0.00 (-0.69, 1.63)	0.067	1.93% ± 5.71%	1.14% ± 1.87%	0.00 (-0.49, 1.22)	0.00 (-0.58, 1.25)	0.345
Staphylococcus edaphicus<br>*Staphylococcus saprophyticus*<br>*Staphylococcus pseudoxylosus*	*Sta. edaphicus* Group	1.13% ± 4.54%	0.60% ± 1.94%	2.17% ± 3.80%	1.43% ± 3.16%	3.19 (0.00, 70.4)	1.08 (0.00, 12.1)	0.061	1.31% ± 2.50%	1.68% ± 3.98%	0.00 (0.00, 5.42)	0.00 (-0.51, 24.3)	0.729
Staphylococcus pettenkoferi	*Sta. pettenkoferi*	0.74% ± 1.59%	0.60% ± 2.00%	0.39% ± 0.85%	0.56% ± 1.18%	0.00 (-0.34, 0.00)	0.00 (-0.51, 1.58)	0.446	0.41% ± 1.42%	0.33% ± 0.79%	0.00 (-0.51, 0.00)	0.00 (-0.85, 0.00)	0.310

Supplemental Figures

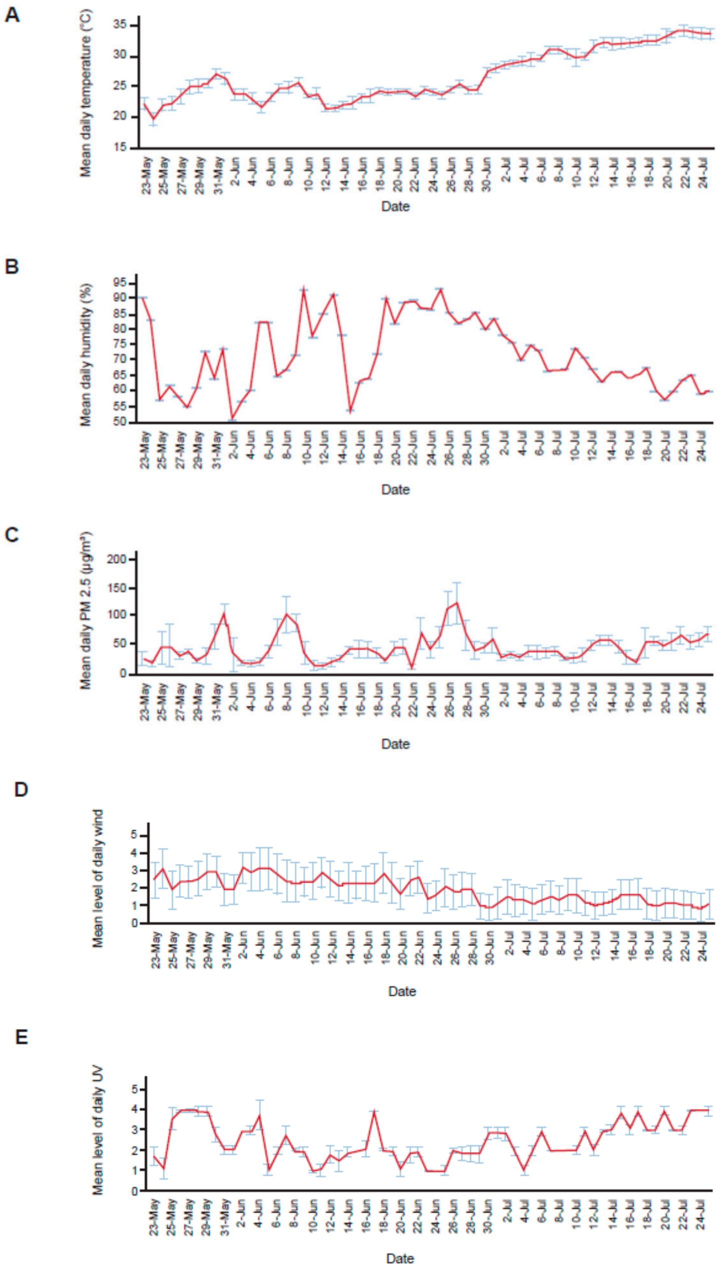

Figure S1 Environmental climate reporting during study period. (A) The mean daily temperature showed an upward trend from May through July, with a mean of 27°C (20–34°C) during the total study period. (B) Mean humidity during the study period was 72% (51–90%). (C) Mean PM2.5 during the study period was 49 μg/m^3 (15–127 μg/m^3). (D) Wind range: level 1–3 (mean: level 2; meteorological standard: 0–12 levels in total, wherein a higher level corresponds to a stronger wind). (E) UV range: level 1–4 (mean: level 2.5; national standard:

5 levels in total; higher level, stronger UV). Abbreviations: PM, particulate matter; UV, ultraviolet.

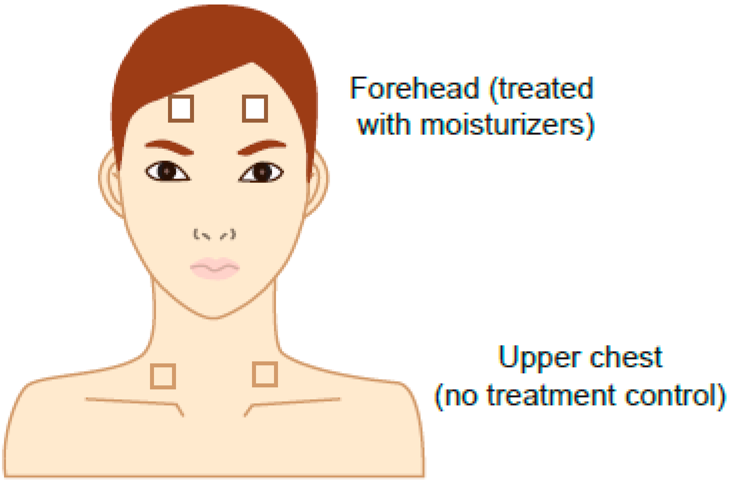

Figure S2 Location of skin evaluations

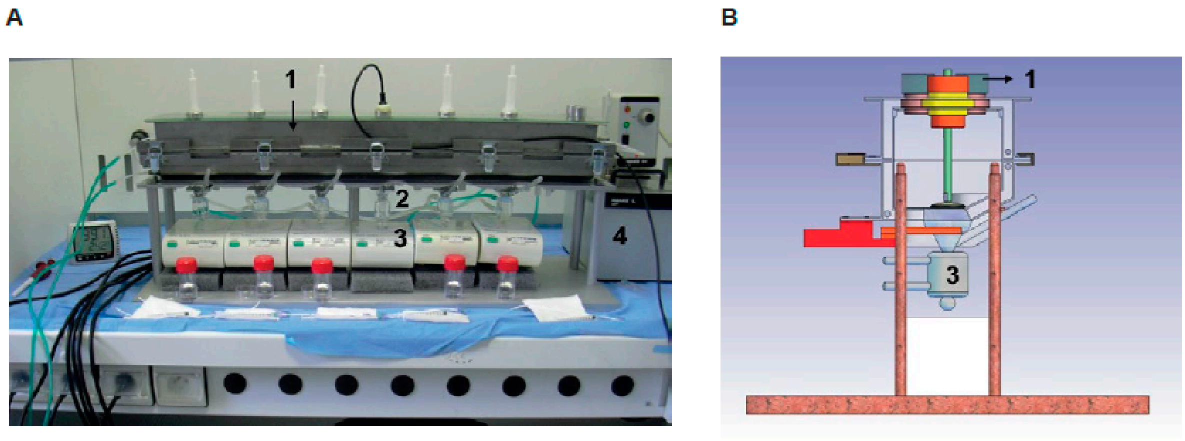

Figure S3 Set-up of the D-SKIN Cell® apparatus. (A) A picture of the set-up showing (1) a stainless-steel chamber with six holes, which is temperature regulated by (2) a water bath. The humidity in the chamber was regulated using saturated salt solutions, which were filled into reservoirs integrated in the chamber. (3) Six diffusion cell receiver compartments were fixed to the chamber, and the temperature of these compartments was regulated with a second water

bath. (4) A sensor measured the temperature and humidity in the chamber. (B) A schematic diagram of a compartment of the D-SKIN Cell®.

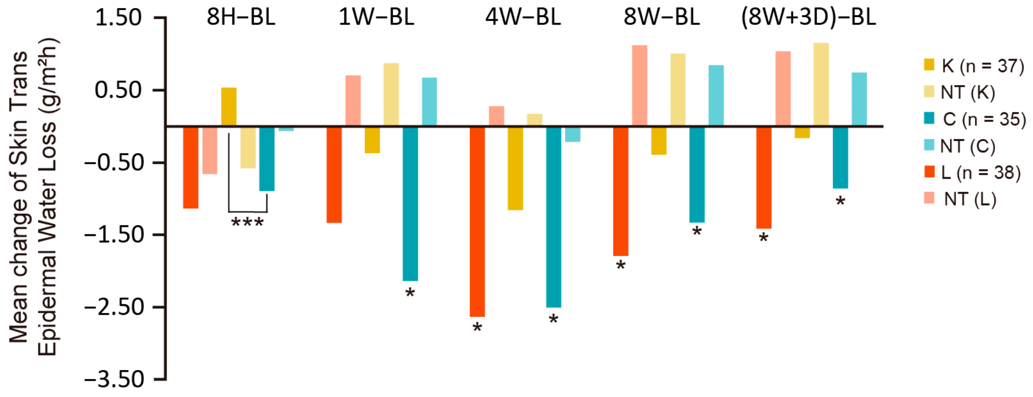

Figure S4 Comparison of skin trans epidermal water loss between the moisturizers/NT. Abbreviations: 8H, 8 hours; 1W, 1 week; 4W, 4 weeks; 8W, 8 weeks; 8W+3D, 8 weeks + 3 days (3 days after treatment was stopped); BL, baseline; K, water gel with yeast extract; C, water gel; L, extra dry emulsion. * means significant difference vs. corresponding NT and $P < 0.05$. *** means significant difference between products and $P < 0.001$. P values were derived using the t-test and ANOVA.

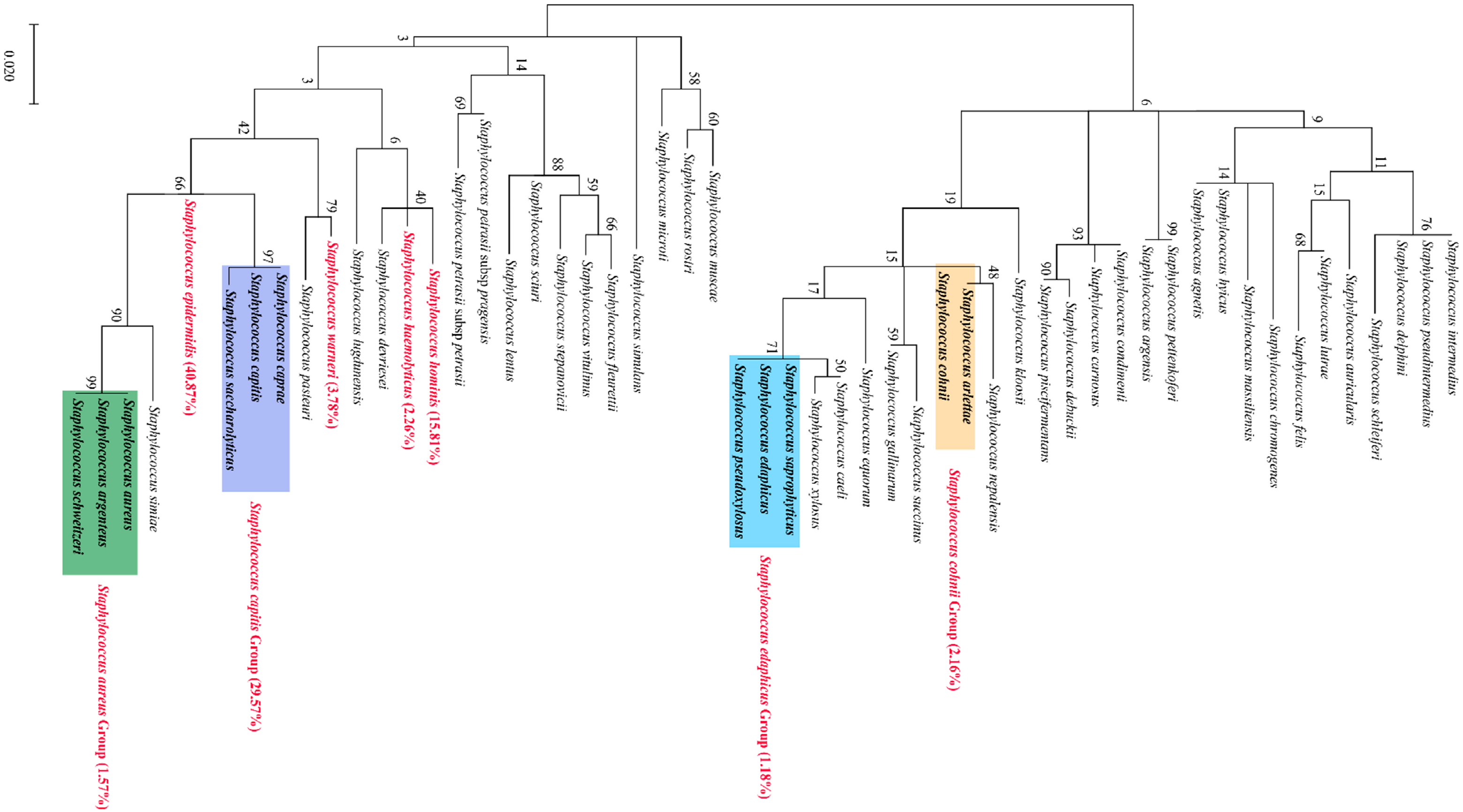

Figure S5 Maximum likelihood phylogenetic tree based on 16S rRNA V1-V2 region sequences of 53 *Staphylococcus* type strains downloaded from the EzBioCloud 16S database (https://www.ezbiocloud.net/resources/16s_download). A K2+G+I model was applied.

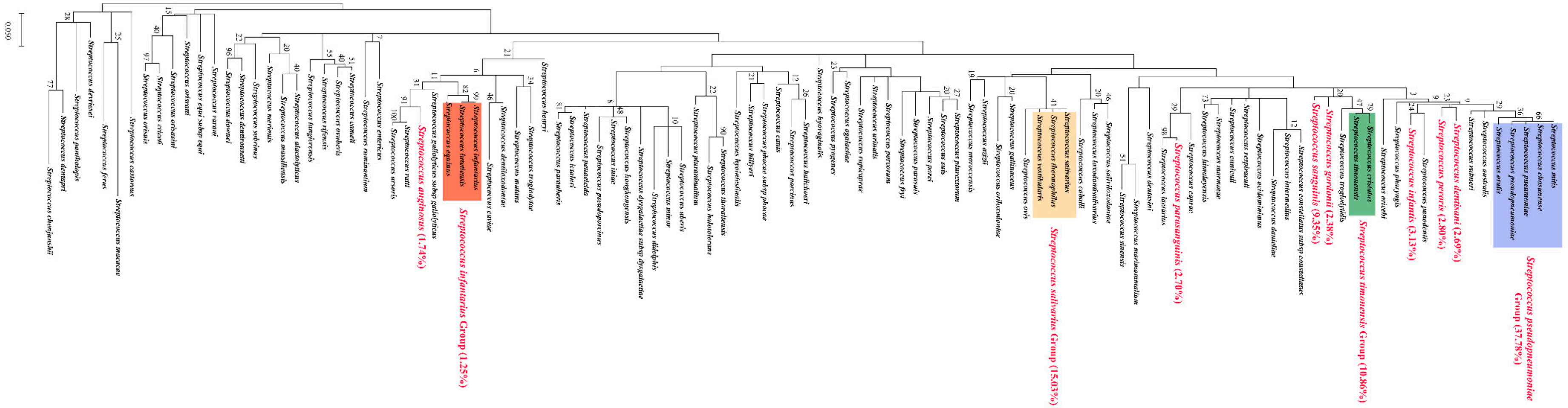

Figure S6 Maximum likelihood phylogenetic tree based on 16S rRNA V1-V2 region sequences of 110 *Streptococcus* type strains downloaded from the EzBioCloud 16S database (https://www.ezbiocloud.net/resources/16s_download). A GTR+G+I model was applied.

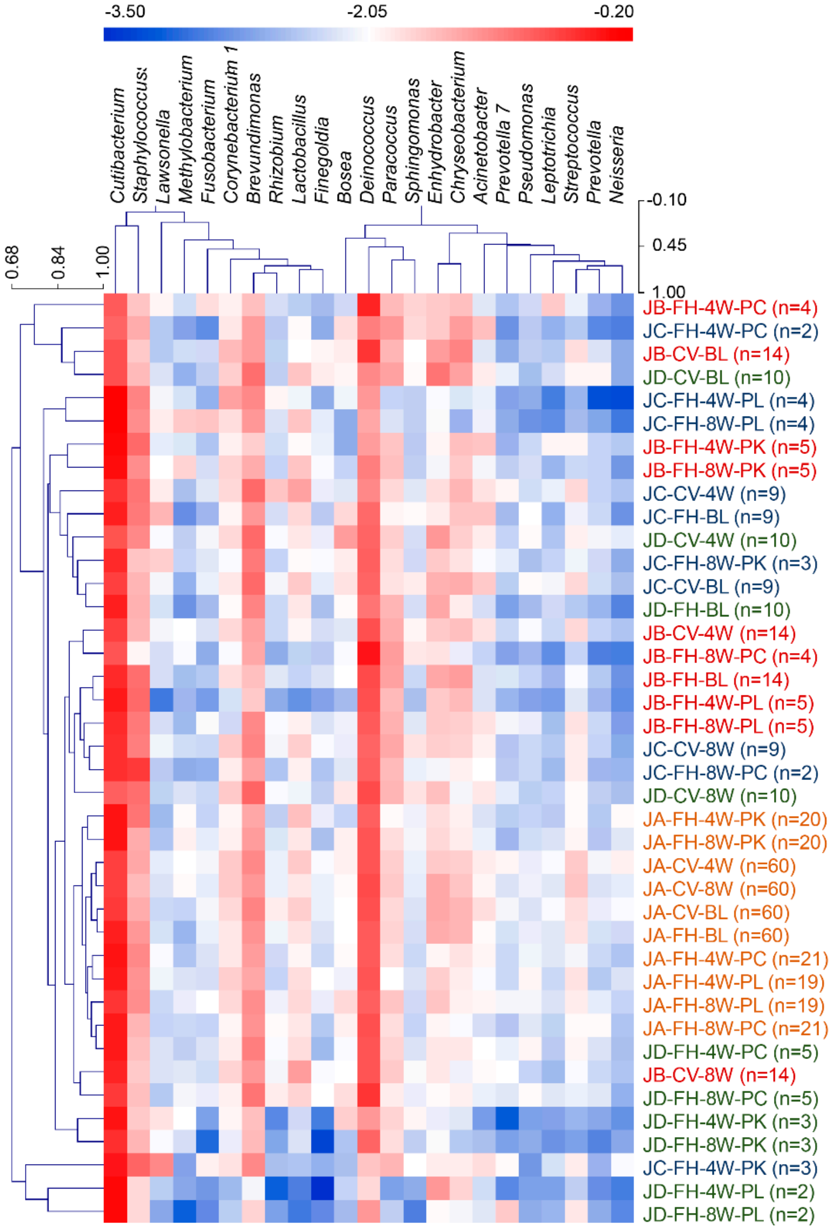

Figure S7 Hierarchical-clustering heat map of 23 major bacterial genera according to 40 groups based on skin sites, sampling times, and occupations. 'JA', 'JB', 'JC', and 'JD' represent subjects' lifestyle of Office worker, Housewife, Student, and Outdoor worker, respectively; 'CV' and 'FH' represent upper chest and forehead, respectively; 'BL', '4W', and '8W' represent sampling at the baseline, 4 weeks, and 8 weeks, respectively. 'PK', 'PC', and 'PL' represent forehead samples applied Moisturizer K (water gel with yeast extract), C (water gel), and L (extra dry emulsion) at 4 and 8 weeks, respectively. Data from four participants with other jobs classified as JE were excluded.

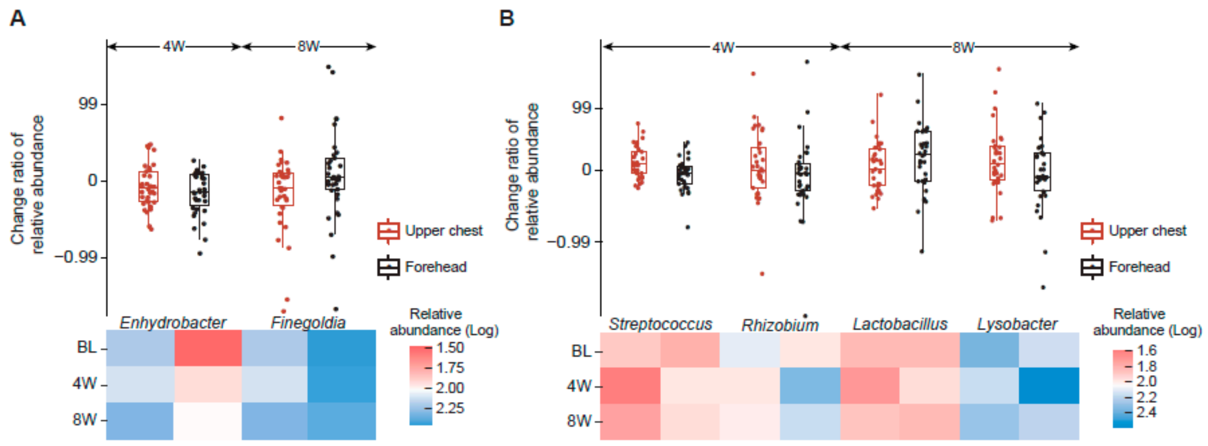

Figure S8 The change ratio of the relative abundance of bacterial genera showed significance at 4 and 8 weeks after using (A) Moisturizer C (water gel) and (B) Moisturizer L (extra dry emulsion). Abbreviations: 4W, 4 weeks; 8W, 8 weeks; BL, baseline.
